# Research Progress on the Application of Novel Wound Healing Dressings in Different Stages of Wound Healing

**DOI:** 10.3390/pharmaceutics17080976

**Published:** 2025-07-28

**Authors:** Lihong Wang, Xinying Lu, Yikun Wang, Lina Sun, Xiaoyu Fan, Xinran Wang, Jie Bai

**Affiliations:** School of Traditional Chinese Medicine, Beijing University of Chinese Medicine, Number 11 East Section of the North Third Ring Road, Beijing 100029, China; 18242099756@163.com (L.W.); luxinying1999@163.com (X.L.); w2499162867@163.com (Y.W.); lina980925@163.com (L.S.); xfanxiaoyu@163.com (X.F.)

**Keywords:** wound healing, wound dressings, hydrogel, microneedle, electrospun nanofiber, stimuli responsive

## Abstract

The complex microenvironment of wounds, along with challenges such as microbial infections, tissue damage, and inflammatory responses during the healing process, renders wound repair a complex medical issue. Owing to their ease of administration, effective outcomes, and painless application, biomacromolecule-based wound dressings have become a focal point in current clinical research. In recent years, hydrogels, microneedles, and electrospun nanofibers have emerged as three novel types of wound dressings. By influencing various stages of healing, they have notably enhanced chronic wound healing outcomes and hold considerable potential for wound repair applications. This review describes the preparation methods, classification, and applications of hydrogels, microneedles, and electrospun nanofibers around the various stages of wound healing, clarifying the healing-promoting mechanisms and characteristics of the three methods in different stages of wound healing. Building upon this foundation, we further introduce smart responsiveness, highlighting the application of stimuli-responsive wound dressings in dynamic wound management, aiming to provide insights for future research.

## 1. Introduction

The skin is the largest organ of the human body, with its primary role being protective. It prevents the invasion of environmental microorganisms and the loss of bodily fluids, while also facilitating sensory perception of the external environment, thermoregulation, and substance exchange [1]. Wounds are disruptions of skin integrity caused by injuries such as trauma, surgery, burns, or diabetic complications. These injuries are often accompanied by impaired physiological functions of the skin, affecting normal human activities. According to the World Health Organization (WHO), burn wounds alone account for over 300,000 deaths annually. Based on healing duration, wounds can be further classified into acute wounds and chronic wounds. Acute wounds have a shorter healing cycle, but the regenerated skin exhibits reduced mechanical strength and may retain minor scarring [2]. Although comprehensive global statistics are lacking, analyses by the American College of Emergency Physicians (ACEP) and surgical databases indicate that as of 2024, annual acute wound management cases (e.g., trauma from accidents, lacerations, and burns, as well as surgical incisions) exceed 10 million in the United States alone [3]. Chronic wounds are characterized by prolonged healing periods, where persistent non-healing or a lack of healing progression compromises the functional restoration of the skin. Chronic wounds are typically categorized into four types based on their etiology and anatomical location: diabetic ulcers, pressure ulcers, venous ulcers, and arterial insufficiency ulcers, with diabetic ulcers being the most prevalent [4,5]. The 2022 report from the International Wound Healing Consortium reveals that over 150 million people worldwide currently suffer from chronic wounds [6], facing risks of amputation, organ loss, or mortality due to suboptimal management and infection exacerbation [7]. Consequently, the substantial increase in demand for wound management among patients has driven significant market expansion. According to Data Bridge Market Research, the global wound healing market (encompassing dressings, devices, biologics, etc.) was valued at USD 18.79 billion in 2023 and is projected to reach USD 25.81 billion by 2031, reflecting a sustained growth trajectory. Wound dressings constitute a substantial segment of this market application.

Wound healing, as a dynamic cascade process, necessitates stage-adaptive dressings to overcome the limitations of conventional therapies. Current clinical interventions (e.g., debridement, negative pressure therapy, and electrotherapy) manage wounds at the macroscopic level but fail to actively modulate pathological microenvironments [8]. Traditional dressings (gauze and cotton pads) provide merely passive isolation due to excessive fluid absorption, adhesion-induced damage, and inadequate microbial barriers [9]. Novel wound dressings enable stage-specific interventions via hierarchical functional designs. For instance, hydrogel dressings achieve efficient hemostasis by absorbing exudate through dynamic hydration, maintaining optimal wound moisture (60–90%) to accelerate epidermal cell migration compared to dry environments [10]. Microneedle arrays penetrate bacterial biofilms, inhibiting inflammation initiation [11]. Electrospun nanofibrous scaffolds mimic extracellular matrix (ECM) topology, significantly enhancing fibroblast proliferation rates [12]. Furthermore, these novel dressings incorporate smart drug delivery systems that release bioactive molecules (e.g., antimicrobials, anti-inflammatory agents, and pro-angiogenic growth factors) in a stage-programmed manner to optimize healing outcomes. Despite extensive research on novel dressings, there is a lack of systematic summaries of these dressings and comprehensive introductions to their healing mechanisms, particularly with regard to the application characteristics and advantages of different stages of wound healing. This review aims to address these knowledge gaps by systematically analyzing representative novel dressings and their pro-healing mechanisms in combination with the different stages of wound healing, thereby providing evidence-based guidance for future clinical selection.

Our discourse commences with an overview of pathophysiological mechanisms across wound healing phases. Subsequently, based on the current research progress, we combine the preparation methods, classification, and applications of the three well-studied novel wound dressings, namely hydrogels, microneedles, and electrospun nanofibers, describing the healing-promoting mechanisms and characteristics of the three dressings in different stages of wound healing. On this basis, we further introduce smart responsiveness, highlighting the application of stimuli-responsive wound dressings in dynamic wound management. Finally, we summarize the main points of this paper and discuss the potential challenges and future prospects of novel wound dressings.

## 2. Hydrogels

Hydrogels are highly hydrophilic three-dimensional (3D) polymer networks that swell rapidly in water and retain a large volume without dissolving. Their physical and chemical properties can be modified to incorporate hemostatic, antibacterial, and anti-inflammatory components and tailored to meet various conditions [13]. Consequently, they exhibit characteristics such as skin-like elasticity; the ability to absorb wound exudate, a porous structure that allows gas exchange, inhibiting bacterial infection while promoting cell proliferation and migration; and the capacity to maintain a moist environment to support the autolytic removal of wound debris, making them widely used in biomedical applications such as wound healing [14], cartilage repair [15], nerve tissue regeneration [16], and the slow release of anti-cancer drugs [17].

### 2.1. Preparation

Preparation of hydrogels can be categorized into physical cross-linking, chemical cross-linking, and heterogeneous cross-linking. Physical cross-linking refers to the formation through non-covalent bonding interactions. This interaction is usually reversible, so it is also known as a pseudogel. Chemically cross-linked hydrogels are polymers that are connected by the addition of a cross-linking agent to form covalent bonds. The hydrogels obtained by this cross-linking method are permanent, so they are also known as true gels [18]. Enzymatic cross-linking represents a distinctive approach in chemical cross-linking strategies, employing specific enzymes (e.g., transglutaminase (TGase), horseradish peroxidase (HRP), and tyrosinase) to catalyze covalent bond formation (e.g., isopeptide bonds and dityrosine linkages) between polymer side chains [19]. This process constructs three-dimensional hydrogel networks with significantly enhanced biocompatibility. Heterogeneous cross-linking is the use of multiple cross-linking methods in the preparation process to make bi- or multi-layer network hydrogels [20]. The common modes of action and advantages and disadvantages of hydrogels obtained by different preparation methods are shown in Table 1. Although the three preparation methods have their own shortcomings, they can be utilized in different stages of wound healing because they can be used according to the specific situation to avoid the shortcomings and can add other substances to make up for them.

**Table 1:** The cross-linking methods and characteristics of hydrogels.

	Mode of Action	Case Studies	Advantages	Disadvantages	Ref.
Physical cross-linking	Ionic interactions	Ca^2+^–alginate hydrogel	Self-healing/stimulus responsive/low toxicity/low cost/simple to operate	Low mechanical strength/poor stability	[21,22,23,24,25,26,27,28]
	Hydrophobic interactions	Methyl acrylate (MA)—(3-acrylamidophenyl)boronic acid (AAPBA) hydrogel			
	Hydrogen bonding	Hydrogel formed by polyurethane containing imidazolidinyl urea (IU), poly (ethylene glycol) (PEG), and methylene diphenyl 4,4-diisocyanate (MDI)			
	Van der Waals forces	Collagen hydrogel			
Chemical cross-linking	Schiff base reaction	Oxidized hyaluronic acid (OHA)–carboxymethyl chitosan (CMCS) hydrogel	High structural stability/mild reaction conditions/tunable drug release properties	Some cross-linkers are cytotoxic	[29,30,31,32,33]
	Michael addition reaction	Hydrogel formed by dopamine-grafted sodium alginate and 4-arm polyethylene glycol tetra-thiol			
	Click chemistry	Hydrogel formed by thiolated γ-polyglutamic acid, glycidyl methacrylate-conjugated γ-polyglutamic acid, and thiolated arginine–glycine–aspartate sequences			
	Enzymatic cross-linking	Hydrogel formed by the action of tyrosinase on collagen and tyramine			
Heterogeneous cross-linking	Two or more cross-inking methods	Carboxyethyl chitosan/oxidized sodium alginate composite hydrogel loaded with AgNPs and Zn^2+^	High cross-linking density/strong mechanical properties/flexible selection of raw materials	Complicated handling/possible interactions of multiple substances	[34,35]

In the hemostasis phase, physically cross-linked hydrogels exhibit stimuli-responsive properties, enabling rapid gelation in response to temperature, pH, or ionic strength changes in the wound microenvironment, thereby absorbing exudate and sealing the wound. However, extreme conditions (e.g., hypothermia or hyperosmotic wounds) may inhibit gel formation. To address this, multi-physical cross-linking strategies combining temperature-responsiveness and ionic interactions can be employed, as exemplified by gelatin (Gel)–alginate composite hydrogels [36]. Additionally, physically cross-linked hydrogels demonstrate self-healing capabilities, minimizing secondary tissue damage, making them suitable for superficial or exudative wounds. Nevertheless, their mild physical interactions result in low mechanical strength, rendering them susceptible to disruption by blood flow or tissue movement, thus limiting efficacy in severe hemorrhage. Consequently, researchers have incorporated nanofibers (e.g., cellulose nanocrystals) to enhance mechanical properties [37]. Chemically cross-linked hydrogels formed via covalent bonds possess high mechanical strength and structural stability, enabling resistance to hemodynamic shear forces and long-term maintenance of hemostatic barriers to prevent rebleeding, making them ideal for deep wounds or massive hemorrhage. Notably, photo-cured or enzyme-cross-linked hydrogels (e.g., photocurable HA hydrogels [38]) allow precise in situ molding. However, residual cross-linkers (e.g., glutaraldehyde) may impair coagulation or induce tissue toxicity, prompting the adoption of biocompatible alternatives such as genipin [39]. Hybrid cross-linked systems integrate two or more cross-linking modalities, for instance, utilizing physical cross-linking in the outer layer for rapid hemostasis and chemical cross-linking in the inner layer to enhance stability. However, their fabrication requires meticulous control of cross-linking sequences and ratios to construct hierarchical networks, increasing process complexity.

In the antibacterial phase, physically cross-linked hydrogels encapsulate antimicrobial agents (e.g., silver ions and antibiotics) via weak interactions, enabling rapid release for immediate bactericidal effects. However, this burst release may lead to insufficient long-term efficacy and potential antibiotic resistance. To overcome this, strategies such as photothermal therapy (PTT) (e.g., nano-silver-loaded hydrogels with near-infrared responsiveness [40]) or multi-layer loading systems have been developed, where the outer layer provides rapid antibiotic release while the inner layer sustains antimicrobial peptide delivery for prolonged efficacy [41]. Chemically cross-linked hydrogels covalently immobilize antimicrobial agents (e.g., quaternized chitosan), enabling sustained release for persistent antimicrobial activity. Nevertheless, a high cross-linking density may impede the diffusion of antimicrobial components to deep infected regions. To address this, researchers have incorporated MMP-sensitive bonds to achieve targeted antimicrobial release in response to infection microenvironments [42]. Hybrid cross-linked hydrogels can achieve temporally controlled antimicrobial action through stratified loading of antibacterial agents. However, careful selection of components is critical to avoid interference with antimicrobial efficacy or unintended immune activation due to compositional complexity.

During the anti-inflammatory and antioxidant phases, physically cross-linked hydrogels readily incorporate natural antioxidants (e.g., gallic acid and resveratrol) through hydrogen bonding or electrostatic interactions, enabling rapid ROS scavenging. However, their weak binding mechanisms may lead to antioxidant leakage due to exudate washout, resulting in inadequate protection against prolonged oxidative stress. To mitigate this, microencapsulation techniques (e.g., liposomes or nanoparticles) can shield antioxidants, or pro-drug formulations can be employed to release antioxidants in response to ROS concentration gradients. Chemically cross-linked hydrogels covalently conjugate antioxidants to ensure prolonged cellular protection against oxidative damage, or they can be functionalized with anti-inflammatory peptides/antibodies for targeted suppression of excessive inflammation. However, covalent grafting may alter the molecular structure of antioxidants or anti-inflammatory agents (e.g., polyphenol oxidation). This challenge can be addressed by employing mild conjugation strategies such as click chemistry to preserve bioactive integrity [43].

In the tissue regeneration phase, the reversible networks of physically cross-linked hydrogels facilitate cell infiltration and ECM remodeling. These hydrogels exhibit gradual degradation synchronized with tissue growth, preventing mechanical constriction. However, excessively rapid degradation should be avoided to maintain adequate scaffolding functions [44]. Chemically cross-linked hydrogels can be grafted with growth factors (e.g., VEGF and EGF) to promote lineage-specific tissue differentiation [45]. Yet, their high cross-linking density and slow covalent network degradation may restrict cell adhesion/proliferation and hinder neo-tissue replacement. Consequently, researchers often utilize degradable cross-linkers to tune degradation kinetics. Hybrid cross-linked hydrogels leverage multiple cross-linking strategies to integrate mechanical gradients, biochemical cues, and electroactivity into hierarchical or composite network architectures, demonstrating enhanced efficacy for tissue regeneration in complex wounds [46].

### 2.2. Classification

Based on material sources, hydrogels can be classified into three categories: natural polymer hydrogels, synthetic polymer hydrogels, and composite polymer hydrogels.

Natural polymer hydrogels include polysaccharides (e.g., chitosan (CS), hyaluronic acid (HA), and sodium alginate (SA)) and polypeptides (e.g., collagen) [47], which exhibit excellent biocompatibility and biodegradability, making them applicable across all phases of wound healing. For example, in the hemostatic stage, the positive charge of CS binds to the negative charge of erythrocytes and accelerates platelet aggregation; collagen activates coagulation factor XII and promotes the generation of thrombin to form blood clots [48]; and SA forms a viscous gel by ion exchange that adheres to wounds to reduce blood loss. In the antimicrobial phase, cations of CS disrupt bacterial membranes, hindering bacterial formation and interacting electrostatically with bacterial membrane lipopolysaccharide (LPS) to increase membrane permeability; SA chelates metal ions (e.g., Cu^2+^) to inhibit bacterial metabolism [49]; and collagen, an excellent carrier of antimicrobial peptides, enhances antimicrobial peptide activity and stability. In the anti-inflammatory and antioxidant phases, SA forms a porous network that absorbs excess exudate, traps TNF-α/IL-6, and reduces the concentration of inflammatory factors; HA inhibits the nuclear factor-κB (NF-κB) pathway and reduces the release of pro-inflammatory factors; in addition, it binds to receptors on the surface of inflammatory cells (e.g., the CD44 receptor), regulates the function of inflammatory cells, and reduces the production of inflammatory mediators [50]. The amino group of CS can reduce ^•^OH/O_2_^•−^, generate stable amino radicals, and reduce the concentration of ROS; the proline residues of collagen can repair oxidative damage; in the tissue regeneration stage, collagen gels can mimic the ECM, guide fibroblasts to infiltrate and promote angiogenesis, and provide an adequate supply of nutrients and oxygen to the regeneration of tissues; the low-molecular-weight fragment of HA will activate the CD44 receptor and promote endothelial cell proliferation [51]. However, their low mechanical strength and susceptibility to proteolytic degradation in wounds necessitate frequent replacement, limiting applications in active wound areas. Thus, modifications such as grafting, cross-linking, or blending with other composites are employed to enhance their mechanical properties [52].

Synthetic polymer hydrogels, such as polyvinyl alcohol (PVA), PEG, polycaprolactone (PCL), carboxymethyl cellulose (CMC), and polyacrylamide (PAAm) [47], form stable networks via cross-linking. They exhibit tunable water absorption, physicochemical properties, and consistent mechanical performance, making them suitable for different stages of wound healing. During hemostasis, PVA forms a stable barrier via hydrogen-bonded cross-linking to resist mechanical stress in dynamic wounds, while CMC dynamically adjusts fluid absorption based on exudate volume to maintain a moist microenvironment. In the antibacterial phase, PAAm networks effectively encapsulate silver nanoparticles [53] or antibiotics; PEG’s low protein adsorption minimizes bacterial adhesion; and PCL’s hydrophobic chains prolong antimicrobial release for sustained efficacy. For anti-inflammatory and antioxidant functions, polyacrylic acid (PAA) can be grafted with antioxidants for persistent ROS scavenging, whereas PVA hydrogels physically isolate external irritants (e.g., bacteria and contaminants) to mitigate secondary inflammation. In tissue regeneration, PCL hydrogels provide high mechanical strength; for example, the compression modulus of hydrogel after adding PCL can reach 12.3 ± 1.5 kPa, which can provide stable mechanical support for cell proliferation and migration [54]. However, potential residual small molecules from synthesis and inflammatory degradation byproducts remain challenges, driving ongoing structural optimization to enhance functionality [55].

Composite polymer hydrogels combine natural and synthetic polymers through physical or chemical methods, synergistically addressing their individual limitations. For example, during hemostasis, CS-PVA composite systems combine electrostatic interactions with mechanical occlusion to achieve effective hemostasis [56]. In antibacterial applications, SA–polydopamine (PDA) hybrid gels enable localized hyperthermia under near-infrared irradiation for bacterial eradication. For anti-inflammatory and antioxidant functions, HA-PBA/PVA hydrogels release anti-inflammatory drugs in response to ROS gradients [57]. In tissue regeneration, HA–silk fibroin (HA-SF) composite hydrogels mimic the porous hierarchical architecture of the native ECM to facilitate complex tissue repair [58]. However, the fabrication of such hydrogels often involves intricate processes, such as the homogeneity challenge of multi-component mixing, the difficulty of synergistic control of multiple cross-linking modes, and the coupled regulation of microstructure and properties, which may lead to batch-to-batch variability, and the long-term biosafety of multi-component systems in cutaneous applications requires further validation.

Based on specific application requirements and material design strategies, hydrogels can be engineered to exhibit tailored functionalities, including injectability, self-healing capability, stimuli-responsiveness, antimicrobial activity, and electrical conductivity. Furthermore, multifunctional composite hydrogels integrating these properties synergistically are achievable through advanced fabrication approaches.

Injectable hydrogels achieve sol–gel transitions via dynamic cross-linking, making them particularly suitable for large irregular wounds as they can precisely release drugs, especially in the hemostatic stage, which can close the bleeding point and rapidly stop bleeding. Zhang et al. [59] developed a HA-based injectable hydrogel by incorporating F127DA nanomicelles, which can shorten the gelation time and improve the hemostatic performance and mechanical stiffness at the same time. This system was successfully applied in cell-laden bioprinting and wound sealing. Its key advantage is avoiding secondary surgical trauma, but balancing gelation kinetics and cell viability remains a challenge.

Self-healing hydrogels repair damage through dynamic covalent bonds or physical interactions, adapting to the mechanical stress of dynamic trauma during tissue regeneration and maintaining long-term scaffold function. However, their self-healing efficiency is sensitive to environmental factors like temperature and humidity. Ding et al. [60] designed a collagen/PVA composite hydrogel using dynamic disulfide bonds, achieving >90% self-healing efficiency at room temperature. Loaded with silver sulfadiazine, it demonstrated synergistic antibacterial effects and accelerated full-thickness skin defect healing in rats. While advantageous for joints and other dynamic regions, reconciling mechanical strength with rapid self-healing remains a hurdle.

Stimuli-responsive hydrogels sense microenvironmental changes (e.g., pH and ROS levels) to trigger functional switching and are particularly suitable for the anti-inflammatory and antioxidant phases; they dynamically regulate the inflammatory response and avoid excessive anti-inflammatory inhibition of regeneration. For instance, phenylboronic acid (PBA)-modified hydrogels release VEGF under high ROS conditions in diabetic wounds to promote angiogenesis [61]. pH-sensitive hydrogels deliver antibiotics in infected wounds (pH > 7.4) and sustain growth factor release during healing (pH ≈ 6.5), enabling stage-specific therapy [1]. However, integrating multiple response logics remains technically challenging.

Bacterial infection is a major impediment to wound healing. Antibacterial hydrogels typically incorporate metal nanoparticles, antibiotics, or natural antimicrobial agents (e.g., CS) to inhibit biofilm formation. However, prolonged antibiotic use may induce resistance, while metal ions (e.g., silver) exhibit cytotoxicity despite broad-spectrum efficacy. For example, silver ions impair fibroblast viability [62]. To address this, CS–silver composites minimize free ion release via electrostatic interactions, enhancing cell survival rates [63].

Conductive hydrogels integrate conductive polymers (e.g., polypyrrole) or nanomaterials (e.g., graphene) to mimic bioelectrical signals. Electrical stimulation (ES) alleviates peri-electrode edema, modulates wound healing-related genes, and enhances fibroblast migration and collagen alignment while exerting antibacterial effects. For example, Ma et al. [64] investigated and developed a bioelectronic patch with an integrated magnesium battery with a conductive hydrogel that combines electrochemical stimulation with ion release function. The hydrogel acted as a cathode to regulate the wound microenvironment by stabilizing ES, thereby promoting electrophilic migration of fibroblasts and enhancing the orderly deposition of collagen. It also releases magnesium ions to inhibit bacterial biofilm formation. In animal studies, the patch resulted in a 2-fold increase in wound healing speed and a collagen arrangement that more closely resembled the natural skin structure without secondary tissue damage. This design provides an efficient electrochemical modulation strategy for chronic wound treatment. However, certain conductive materials may induce oxidative stress with unverified long-term stability. For instance, conductive polymers like polyaniline (PANI) and polythiophene often require acidic processing media and small-molecule dopants during dressing fabrication. While these dopants critically govern biocompatibility and antibacterial activity, long-term implantation can trigger significant oxidative stress, thereby inducing apoptosis and inflammatory responses [65].

### 2.3. Application in Different Stages of Wound Healing

#### 2.3.1. Hemostasis

Hemostasis, the inaugural phase of wound healing, serves as a critical metric for dressing efficacy. Hydrogel dressings demonstrate exceptional hemostatic capabilities through multifaceted mechanisms. The hemostatic performance arises from synergistic interactions between physicochemical properties and coagulation cascades. The macroporous architecture enhances the blood contact surface area while forming mechanical barriers at bleeding sites. Molecular dynamics simulations reveal that electrostatic interactions between hydrogel amino/carboxyl groups and tissue proteins not only achieve mechanical sealing but also activate platelet integrin αIIbβ3 signaling, accelerating fibrinogen cross-linking [66]. CS-based hydrogels exemplify this mechanism, where protonated amino groups significantly enhance platelet aggregation rates [67]. Alginate hydrogels conversely accelerate thrombin generation via Ca^2+^-mediated Factor XII activation, reducing clotting time [68]. However, excessive adhesion risks secondary injury, which is addressed through pH-responsive catechol moieties that modulate adhesion strength according to wound pH, enabling controlled detachment [66]. In addition, loading hydrogels, as a matrix material with drugs having hemostatic activity, can better inhibit bleeding. Guo et al. [69] developed QCS/TA hydrogels where TA-induced vasoconstriction reduced the bleeding time by 42% in murine models. However, prolonged residence may impede granulation tissue formation, necessitating optimized degradation kinetics.

#### 2.3.2. Antibacterial

**Figure 1:**
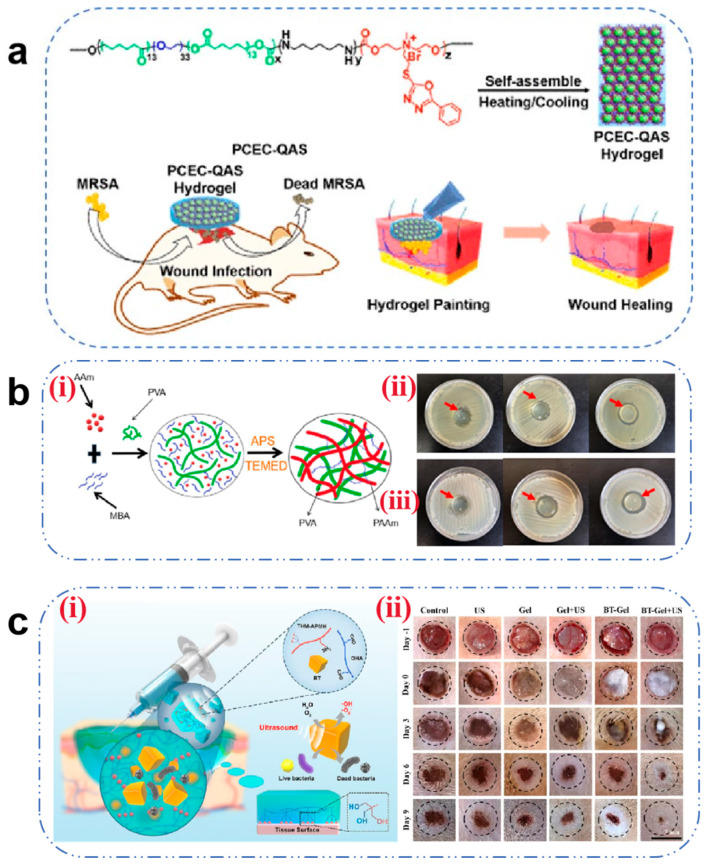
(**a**) Schematic diagram of the antibacterial hydrogel for the treatment of wounds infected with methicillin-resistant *Staphylococcus aureus* [71]. Copyright 2016, ACS Publications. (**b**) (**i**) Schematic diagram of the formation process of PVA/PAAm IPN hydrogels. (**ii**) Antibacterial effect of different concentrations of PHMG hydrogels on *E. coli*. (**iii**) Antibacterial effects of different concentrations of PHMG hydrogels on *S. aureus*. The concentration of PHMG from left to right is 0%, 0.01%, and 0.1%, respectively [72]. Copyright 2023, Elsevier. (**c**) (**i**) Schematic diagram of the ultrasound-triggered piezoelectric composite hydrogel for infected wound treatment. (**ii**) Representative photographs of infected wounds at different times during the treatment. Scale bars: 7 mm [77]. Copyright 2023, Elsevier.

Microbial infection remains a predominant obstacle to wound healing, as bacterial colonization perpetuates inflammatory responses and delays recovery. This underscores the imperative for antimicrobial hydrogel development. Cationic hydrogels (e.g., quaternized CS and polycationic peptides) exert bactericidal effects through electrostatic attraction to negatively charged bacterial membranes, inducing membrane destabilization and cytolysis [70]. Liu et al. [71] engineered a self-assembling antimicrobial hydrogel via chemical conjugation of oxadiazole-functionalized QAS with PCEC copolymers. The resultant PCEC-QAS nanoparticles spontaneously organized into irreversible hydrogels (Figure 1a), demonstrating broad-spectrum efficacy, particularly against wound healing of MRSA infection. However, irreversible hydrogels often fail to accommodate the dynamically evolving wound microenvironment and may impose mechanical constraints on neo-tissue expansion during late-stage healing. Consequently, the development of dynamic covalent hydrogels is recommended to achieve adaptive responsiveness to wound deformation.

Alternative antimicrobial strategies have emerged to combat antibiotic resistance. Incorporation of non-antibiotic biocides into hydrogels presents a viable solution [14]. Li et al. [72] fabricated PVA/PAAm IPN hydrogels via one-pot radical polymerization by integrating PHMG polymer biocides (Figure 1b). This design significantly enhanced bactericidal activity against both Gram-positive and Gram-negative pathogens. As researchers continue to explore this field, metal nanoparticles (Ag, Cu, and Fe) have gained prominence as alternative antimicrobials. Zhao et al. [73] developed a dual-cross-linked COC hydrogel where in situ reduction of Ag^+^ to AgNPs achieved potent antibacterial effects. The released Ag^+^ ions penetrate bacterial membranes, binding thioredoxin reductase to disrupt respiratory chains and induce apoptosis. Despite their efficacy, uncontrolled ion release kinetics may cause mitochondrial DNA damage and suppress ATP synthesis [74]. Advanced strategies employ MOF encapsulation to modulate Ag^+^ release rates (67% reduction) while preserving antibacterial potency [75].

Stimuli-responsive systems integrating hydrogels with physical fields (acoustic, optical, electrical) enable spatiotemporal drug control and multimodal antimicrobial action. PTT employs photothermal agents (AgNPs, CuS, and GO) to generate localized hyperthermia (ΔT = 42–50 °C under 808 nm NIR), inducing membrane lipid phase transition, DNA denaturation, and controlled antibiotic release. Gao et al. [76] demonstrated a significant enhancement in vancomycin release via NIR-responsive systems. Piezocatalytic therapy utilizes piezoelectric materials (e.g., BaTiO_3_) to generate ROS under ultrasound. Liu et al. [77] designed a multifunctional composite hydrogel, BTOHA/THM-APMH (BT-Gel) (Figure 1c), which can rapidly increase the ROS concentration in a short time under US irradiation, achieve broad-spectrum bactericidal properties by oxidizing the bacterial membrane phospholipids and breaking DNA strands, and promote the healing of wounds under bacterial infection. However, challenges persist in clinical translation, including low catalytic efficiency and off-target effects [78].

#### 2.3.3. Anti-Inflammatory and Antioxidant

**Figure 2:**
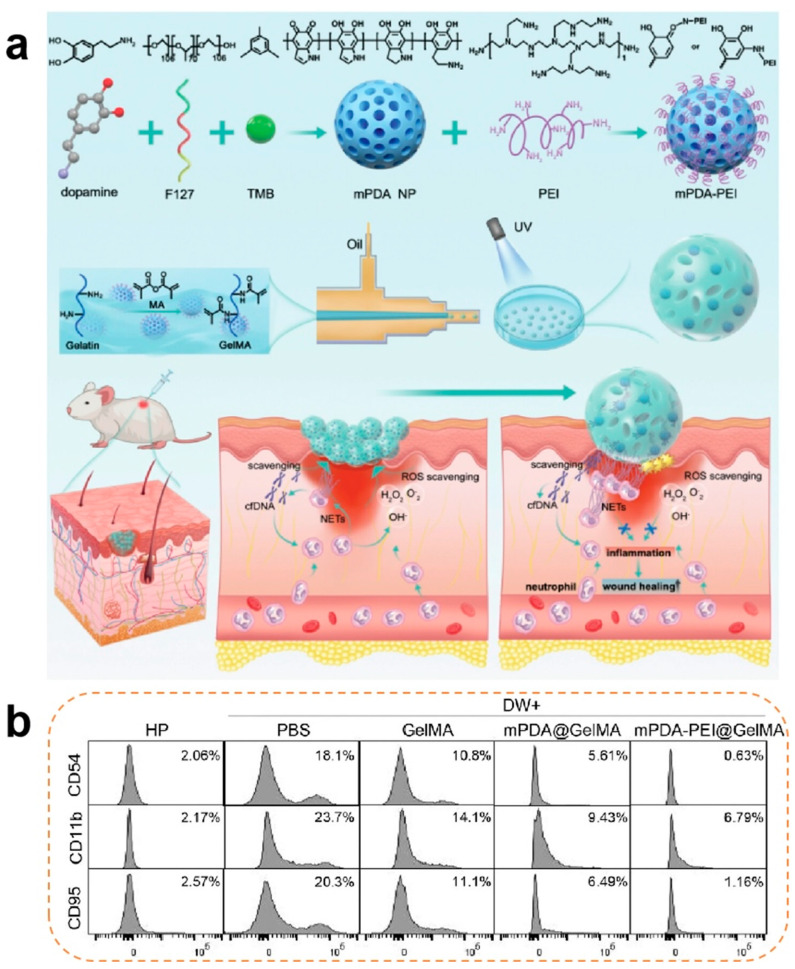
(**a**) Synthesis process of neutrophil extracellular traps (NETs) and scavenger ‘micro-cage’ mPDA-PEI@GelMA along with its application in treating wounds in diabetic mice through the NET scavenging strategy. NETs were introduced into the ‘micro-cage’ along with wound exudation, and the cationic mPDA-PEI immobilizes them inside the ‘micro-cage’ through a robust binding affinity to the cfDNA web structure. (**b**) Effects of diabetic wound exudation on the expression of CD95, CD54, and CD11B in healthy human neutrophil cells, along with the treatment effects of mPDA-PEI@GelMA [80]. Copyright 2024, Wiley-VCH GmbH.

Anti-inflammatory modulation constitutes the cornerstone of the proliferative phase. Persistent M1 macrophage polarization coupled with elevated pro-inflammatory cytokines (IL-6 and TNF-α) and ROS levels perpetuates inflammatory cascades, impeding healing progression. Anti-inflammatory hydrogels address this through radical scavenging, chemokine sequestration, and macrophage phenotype reprogramming [79]. Xiao et al. [80] proposed the addition of methacrylic acid (MA) to a gel solution for cross-linking to form GelMA microspheres, which were then combined with cationic polyethyleneimine (PEL) and functionalized mesoporous polydopamine (mPDA) to develop mPDA-PEL@GelMA. mPDA-PEL@GelMA was shown to be effective in the treatment of diabetic wounds through the scavenging of ROS and enhancement of binding to cfDNA to inhibit neutrophil extracellular traps (NETs), resulting in a significant reduction in the proportion of M1-type macrophages in diabetic wounds. The material also upregulated IL-10 expression and promoted macrophage polarization towards the M2 type (Figure 2).

Persistent inflammatory conditions lead to substantial accumulation of ROS, which in turn exacerbates inflammatory responses through positive feedback loops. Elevated ROS levels not only induce cellular and DNA damage but also impair angiogenesis, thereby prolonging the wound healing process [81]. Maintaining redox homeostasis is therefore critical for tissue regeneration. The design of antioxidant hydrogels predominantly relies on the incorporation of exogenous antioxidants. Table 2 summarizes the commonly employed antioxidants in hydrogel systems, along with their characteristics and mechanisms. Current fabrication strategies encompass two approaches: (1) direct encapsulation of antioxidants and (2) covalent grafting of antioxidant moieties onto polymer backbones. While direct loading offers simplicity in preparation, it often suffers from burst release kinetics and compromised stability. In contrast, grafting methods enable sustained antioxidant activity but require complex synthetic procedures. Antioxidants selected for conjugation typically possess functional groups such as carboxyl, hydroxyl, aldehyde, or thiol moieties, with thiol-containing compounds and natural polyphenols being widely utilized [82]. Inorganic nanoparticles (e.g., cerium oxide and selenium) are generally incorporated via physical entrapment. Despite their efficacy, concerns regarding long-term stability and potential cytotoxicity necessitate rigorous biocompatibility evaluations [82].

**Table 2:** Antioxidant components in hydrogels.

Category	Antioxidant Component	Specificities	Antioxidant Mechanism	Ref.
Natural polyphenols	Curcumin	Significant antioxidant properties/poor water solubility/poor stability	Engaging in hydrogen atom transfer mechanisms to quench ROS/RNS	[83]
	Tannic acid (TA)	Biocompatible/multiple binding sites/toxic at high concentrations		[84]
	Protocatechuic acid (PCA)	Easily grafted		[85]
	protocatechuic aldehyde (PA)	Biocompatible/easy to form reversible covalent bonds		[86]
	Gallic acid (GA)	Poor stability		[87]
	Epigallocatechin-gallate (EGCG)	High antioxidant properties/low oral bioavailability		[88]
	Polydopamine (PDA)	Significant antioxidant properties/anti-inflammatory		[89]
Thiol-based compounds	Glutathione (GSH)	Good water solubility/poor stability	Thiol groups engage with various free radicals, thereby mitigating oxidative stress-induced cell damage	[90]
	Alpha-lipoic acid (ALA)	Can act synergistically with a variety of antioxidants		[91]
Inorganic nanoparticles	Fullerenol nanoparticles	Biocompatible	Forms strong bonds with ROS and destroys ROS by electron transfer	[92]
	MoS_2_ nanoparticles	High photo-thermal conversion rate/antibacterial properties	Similar natural antioxidant enzymes to reduce ROS	[93]
	CeO_2_ nanoparticles	Easy to synthesize/biocompatible		[94]
	Platinum nanoparticles	Wide range of oxidation resistance/good environmental adaptability/high cost		[95]
	Metal–organic frameworks (MOFs)	High specific surface area/porosity/degradable structure/excellent catalytic activity/reliability		[93]

Consequently, hydrogels fabricated from intrinsically antioxidant materials, including fucoidan, CS, lignin, polypeptide chains, polyaniline, sodium hyaluronate, PVA, and PBA, have gained prominence due to their inherent radical-scavenging capabilities [96]. Zhao et al. [97] engineered a smart bilayer hydrogel (Dual-Gel) using sodium hyaluronate, which achieves spatiotemporal regulation of biphasic ROS management. During the infectious phase, functionalized nanovesicles (MTB@ANVs) are released to elevate ROS levels for bacterial eradication, while the proliferative phase leverages hyaluronate-mediated ROS scavenging to facilitate tissue regeneration.

It should be noted that the distinction between intrinsically antioxidant hydrogels and antioxidant-loaded systems is not absolute, as many self-antioxidative matrices can be further augmented with additional free radical scavengers.

#### 2.3.4. Tissue Regeneration

**Figure 3:**
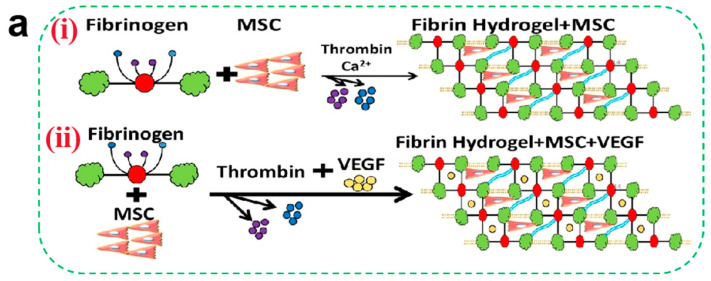

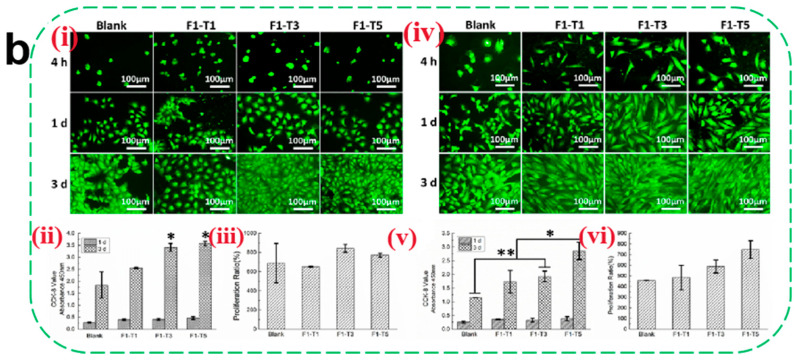
(**a**) (**i**) Schematic of encapsulated BMSCs in fibrin gels. (**ii**) Schematic of encapsulated VEGF and BMSCs in fibrin gels. (**b**) Evaluation of epithelial cell compatibility. Morphology of EC (**i**) and SMC (**iv**) of different fibrin gels; (**ii**,**v**) refers to the CCK-8 results, and (**iii**,**vi**) refer to the proliferation ratio of ECs and SMCs, respectively (mean ± SD, *n* ≥ 3, * *p* < 0.05, ** *p* < 0.01) [45]. Copyright 2021, Elsevier.

Tissue regeneration during the later stages of wound healing critically depends on robust vascularization. Angiogenesis—a prerequisite for successful regeneration—requires precise coordination of endothelial cell migration, vascular basement membrane remodeling, and pericyte recruitment (directional migration of pericytes to the neovasculature). Certain hydrogel matrices inherently promote vascularization: CS enhances endothelial cell migration via HIF-1α/VEGF signaling activation, while fibrin facilitates vascular network formation through integrin αvβ3-mediated FAK phosphorylation [98]. Although native hydrogels exhibit modest pro-angiogenic effects, researchers often incorporate functional components (e.g., metal ions and growth factors) to potentiate vascular regeneration. Metal ions such as Zn^2+^, Mg^2+^, and Cu^2+^ demonstrate potent angiogenic properties. Zn^2+^ augments glucose metabolism in osteoblasts via RAC1 modulation to fuel vascularization [99]. Mg^2+^ stimulates endothelial nitric oxide (NO) synthesis, suppresses angiostatin, and upregulates VEGF and bFGF expression [100]. Cu^2+^ stabilizes HIF-1α under normoxic conditions, mimicking hypoxia to enhance VEGF-driven endothelial proliferation and migration. Liu et al. [101] developed a Cu^2+^-CS hydrogel that induces VEGF upregulation via controlled ion release. However, prolonged exposure to high metal ion concentrations may inhibit cell proliferation, necessitating precise delivery kinetics. Alternative strategies involve co-loading growth factors (VEGF and bFGF) or stem cells (ADSCs and BMSCs) into hydrogels. Tan et al. [45] demonstrated that fibrin gels co-encapsulating VEGF and BMSCs (Figure 3a) significantly promoted the adhesion and proliferation of smooth muscle cells and vascular endothelial cells (Figure 3b) through paracrine signaling (a process in which cells secrete biologically active factors that diffuse in the local microenvironment and then act on neighboring target cells to modulate their proliferation, differentiation, or functional expression), leading to an increase in capillary density.

Beyond angiogenesis, tissue regeneration requires spatiotemporal coordination among keratinocytes, fibroblasts, and endothelial cells [102]. Hydrogel-mediated delivery of pro-regenerative components thus offers distinct advantages in orchestrating multicellular interactions. Substance P (SP), a neuropeptide distributed in the central nervous system, plays pivotal roles in cellular proliferation. TGF-β1 serves as the most potent inducer of fibroblast-to-myofibroblast differentiation. Park et al. [103] prepared a CS hydrogel (CSMP-PF) co-delivering SP and TGFβ1, which was effective in inducing fibroblast differentiation and increasing the proportion of α-SMA-positive myofibroblasts, a key cell involved in wound contraction.

However, excessive fibrogenesis must be mitigated to prevent pathological scar formation.

## 3. Microneedles

Microneedles (MNs) are a novel drug delivery system consisting of sharp micron-scale needle arrays that enable drugs to penetrate the skin barrier and reach the target site through microchannels [104]. It has gained widespread attention due to several advantages such as avoiding drug degradation and the first-pass effect, being non-invasive and painless, enabling simple operation, and allowing local control. MNs were originally designed for transdermal drug delivery of drugs that are hindered by factors such as large size, hydrophilicity, poor absorption, and unstable properties. In recent years, they have gradually been applied in wound healing due to their excellent delivery efficacy [105]. Compared to traditional wound dressings, MNs offer good permeability and extremely low invasiveness, allowing them to penetrate microbial biofilms [106] and scabs that form during the healing process, thereby enabling active ingredients to enter healthy cells and exert their therapeutic effects. Furthermore, certain microneedle formulations possess swelling characteristics similar to those of proteins, wherein the needle tips expand upon insertion into the skin, thereby enhancing physical interlocking with adjacent tissues [107].

### 3.1. Classification and Preparation

**Figure 4:**
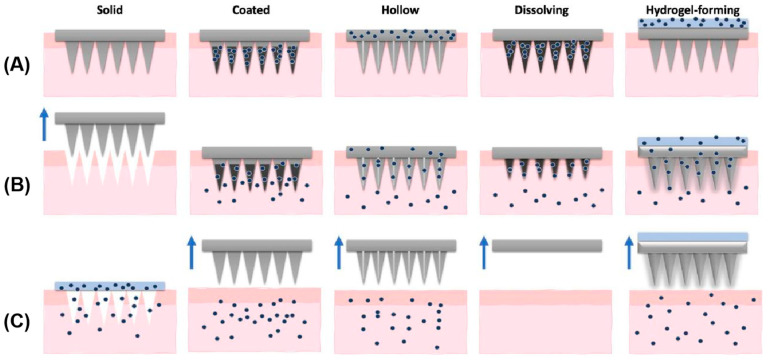
Types of microneedles: solid, coated, hollow, dissolving, and hydrogel-forming. (**A**) Application of MNs; (**B**) drug (dots) release; (**C**) removal of MNs (arrows). An exception is observed for solid MNs, in which the device is removed prior to drug application. Reprinted with permission from ref. [108]. Copyright 2021 Elsevier.

According to different methods of fabrication and action, MNs can be classified into five types: solid, hollow, coated, dissolving, and hydrogel-forming MNs (Figure 4).

Dissolvable MNs primarily encapsulate drugs within dissolvable or biodegradable polymer needles, including CS, HA, CMC, PVA, and polyvinylpyrrolidone (PVP). Upon penetrating the skin, the needle-like polymer structure dissolves, releasing the drug [109]. Most dissolvable MNs are fabricated using traditional methods like microcasting or micromolding, in which raw materials are filled into molds. Researchers have recently introduced technological improvements by utilizing techniques including spray atomization to fill molds, air-blowing methods to shape polymer droplets, and photopolymerization to prepare dissolvable MNs [110]. Although dissolvable MNs have relatively low penetration into the stratum corneum and limited drug loading capacity, they have the advantages of being minimally invasive, causing low tissue damage, offering controlled dissolution rate, allowing simple preparation, and eliminating the need for needle retrieval, making them a type of microneedle frequently used in the field of wound healing.

Hydrogel MNs are typically composed of cross-linked polymers including copolymers of poly(ethylene glycol) methyl ether and maleic anhydride (PMVE/MA), methacrylated hyaluronic acid (MeHA), gelatin methacrylate (GelMA), and PVA [111]. The drug is placed on top of the hydrogel microneedle in the form of a storage layer, and when the needle is inserted into the skin, it quickly absorbs interstitial fluid and swells to form a porous structure, allowing drugs stored at the top of the microneedle array to diffuse into the skin tissue through these micropores [109]. Various methods exist for hydrogel preparation, with the most common involving microfabrication techniques, where physical or chemical cross-linking occurs between molecules to form hydrogels, which are then placed into micro-molds to produce hydrogel MNs [112]. The expansibility of hydrogel MNs enables absorption of excess exudate, thereby reducing bacterial infection at the wound site. Additionally, they have low residual polymer content and high drug loading capacity, making them second only to dissolvable MNs in wound repair applications. However, a hydrogel microneedle also has its drawbacks; the general hydrogel microneedle will have a sudden release effect after the administration of the drug, making it prone to toxic side effects; if the selected polymer does not have biocompatibility with the microneedle, this also makes it prone to causing toxic reactions.

The materials used in the preparation of solid MNs include metals, glass, ceramics, silicon, and polymers [113]. The drug delivery process generally consists of two steps: first, the needle tip penetrates the skin to create hydrophilic microchannels, followed by the diffusion of drugs from local formulations (e.g., gels, creams, lotions, and ointments) or transdermal patches through the microchannels into deeper skin layers [109]. Methods such as 3D printing [114], diffraction lithography [115], etching, and hot embossing [116] are used to fabricate MNs. Solid MNs can only be used to deliver drugs when used for wound healing, and their application is relatively limited due to the cumbersome drug delivery steps and the short opening time of the MNs, which together result in the inability of sustained drug release. However, their high mechanical strength can destroy the biofilm and facilitate the drug to reach the deeper layers of the skin to play a therapeutic role. Omolu et al. [117] developed a microneedle roller to deliver doxycycline to tissue sites via microchannels, inhibiting MMPs activity, which reduces ECM and collagen degradation and lowers bacterial activity, thereby promoting chronic wound healing.

Coated MNs are solid MNs with drug formulations coated on their surfaces. Upon insertion into the skin, the coating layer rapidly dissolves, releasing the drug to the targeted site [118]. The range of drugs that coated MNs can carry is broad, including macromolecules such as proteins, DNA, and vaccines, as well as small molecules and even microparticles that can be coated onto the surfaces of solid MNs [119]. Furthermore, various methods are available for drug coating, including dip-coating, casting deposition, spray drying, and inkjet printing [120,121]. Coated MNs can only be used to deliver drugs when promoting wound repair using solid MNs, but they can release the drug immediately after being applied to the wound, which has a rapid onset of action than solid MNs [109]. However, this also places high demands on the adhesion of the drug to the needle. To address this problem, Fu et al. [122] used iron-coordinated polymer nanowire networks (Fe-IDA NWs) as the microneedle coating and polyethylene glycol diacrylate (PEGDA) as the solid needle, and Fe-IDA NWs were coated onto the surface of PEGDA by UV irradiation to obtain Fe-IDA MNs. The spider web-like Fe-IDA NWs exhibit a specific viscosity, allowing them to adhere well to the microneedle surface during coating, and they demonstrate good enzymatic activity and antibacterial properties, significantly promoting the healing of infected wounds. However, coated MNs have had some but relatively few applications in wound healing due to their inability to better regulate the rate of drug release and their limited surface, which results in a small drug loading capacity.

Hollow MNs are fabricated using the same materials as solid MNs. By integrating a cavity within the needle and incorporating a small hole at the tip, drugs can be released from the cavity reservoir through the microchannel formed by the hole to reach the treatment site [123]. The most common fabrication methods for hollow MNs are photolithography [124], laser cutting [125], 3D printing [126], and metal plating [127]. The main advantages of this type of microneedle include large drug loading capacity and high mechanical strength [128]. However, the hollow tip may be blocked by skin tissues during insertion [109], resulting in fewer applications of hollow MNs as wound dressings, and most of the studies have focused on insulin injection [129], ophthalmic medications [130], vaccine delivery [131], and monitoring [132].

### 3.2. Application in Different Stages of Wound Healing

#### 3.2.1. Hemostasis

**Figure 5:**
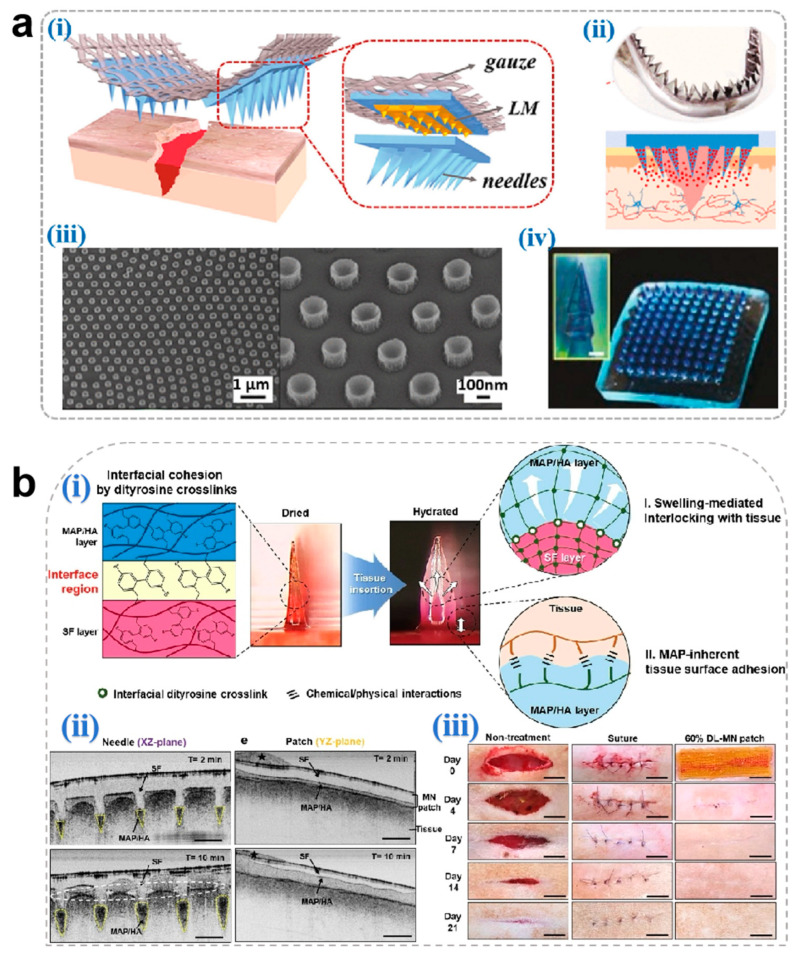

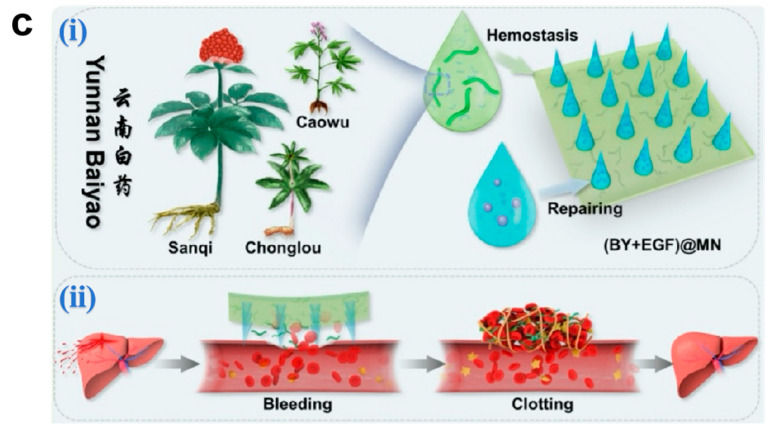
(**a**) Novel microneedle styles: (**i**) an eagle claw-style microneedle, (**ii**) a shark teeth-style microneedle, (**iii**) an octopus nanosuction-style microneedle, and (**iv**) a pagoda-style microneedle [133]. Copyright 2024, Elsevier. (**b**) (**i**) Schematic illustration for the proposed working mechanisms of a hydrogel-forming adhesive MN patch consisting of a MAP-based swellable and sticky shell and an SF-based non-swellable core. (**ii**) Cross-sectional XZ- and YZ-plane images for the in situ swelling behavior of the 60% DL-MN patch following insertion into the wet tissue surface. Selective swelling (yellow dashed region), surface entanglement by water absorption (white dashed circle), and water (black star) are indicated. Scale bar = 500 μm. (**iii**) Macroscopic images for the healing of rat skin wounds after non-treatment, suture treatment, and 60% DL-MN patch treatment during the observation period. Scale bar = 1 cm [136]. Copyright 2021, Elsevier. (**c**) (**i**) A design of (BY + EGF)@MNs with divisional structures. (**ii**) The (BY + EGF)@MNs could exert strong pro-coagulant capacity in a rat hepatic hemorrhage wound model [134]. Copyright 2023, Springer.

Hemorrhage resulting from cutaneous injuries often leads to microbial infection and inflammatory complications when managed with conventional gauze dressings. To address this limitation, MNs have been engineered with specialized geometries to mechanically approximate wound edges and achieve hemostasis through physical sealing (Figure 5a) [133]. Hydrogel MNs [134] and dissolving MNs [135] represent the predominant architectures for such applications.

MNs achieve rapid hemostasis through multimodal mechanisms combining tissue adhesion, mechanical interlocking, and biochemical regulation. Jeon et al. [136] developed a mussel adhesive protein (MAP)-based hydrogel MN system. The MAP outer layer undergoes rapid hydration-induced swelling upon dermal penetration, establishing robust tissue adhesion through mechanical anchoring with collagen fibrils, thereby effectively sealing hemorrhagic sites (Figure 5b). Yang et al. [134] engineered a Yunnan Baiyao-loaded MN patch, where the traditional hemostatic agent achieves rapid dissolution (<6 min) upon wound application. The released components activate coagulation Factor XII and stimulate platelet α-granule secretion, significantly accelerating fibrin cross-linking to achieve hemostasis in murine hepatic hemorrhage models (Figure 5c). Although Yunnan Baiyao exhibits potent hemostatic effects, its complex multi-component composition and significant batch-to-batch variation during preparation may result in inconsistent coagulation efficacy following drug release from microneedles.

Despite their efficacy, challenges persist in precision manufacturing of MN tips. Suboptimal dimensional control or structural deformation during demolding may compromise capillary integrity, exacerbating hemorrhage [135]. Rigorous optimization of tip geometry and mechanical properties is therefore critical to ensure clinical safety.

#### 3.2.2. Antibacterial

During wound healing, a biopolymer layer highly conducive to microbial growth, forms at the wound site, interacting with pathogenic microorganisms to develop bacterial biofilms. These biofilms act as physical barriers that impede drug delivery and induce persistent inflammatory responses [11]. Microneedle arrays can penetrate biofilms, creating microchannels (aperture ≈ needle diameter) to enhance drug permeation efficiency. Hydrogel and dissolvable MNs are predominantly employed for antibacterial purposes, while solid and coated MNs are less frequently utilized.

The antibacterial efficacy of microneedle dressings is closely associated with matrix material selection and drug-loading strategies. In matrix design, natural cationic polymers such as CS are favored for their intrinsic antimicrobial properties. Protonated amino groups in CS disrupt the outer membrane integrity of Gram-negative bacteria via electrostatic interactions, while metal ion chelation blocks bacterial metabolic pathways (e.g., TLR4/NF-κB signaling inhibition) [137].

**Figure 6:**
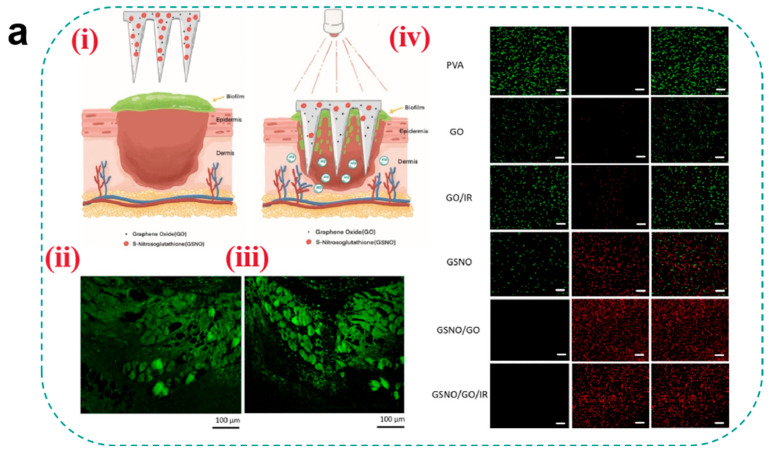

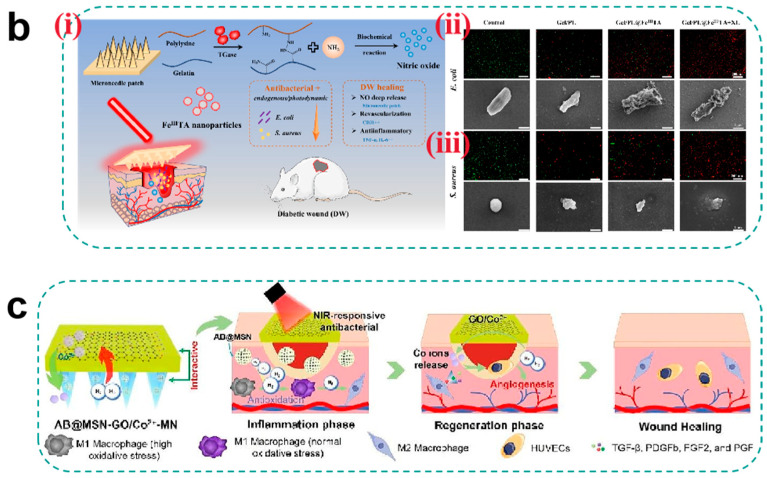
(**a**) (**i**) Schematic of light-regulated NO release from GO-GSNO-HFMNs. GO-GSNO-HFMNs can destroy the biofilm structure, and the NO released by GSNO irradiated with near-infrared light can be regulated for wound healing. (**ii**) Fluorescence image of biofilm. The biofilm was stained with ConA-FITC and monitored under a CLSM. (**iii**) Representative fluorescence image acquired after attachment and removal of GO-GSNO-HFMNs. (**iv**) Confocal microscopy images of MRSA biofilms treated with different groups of HFMNs for 24 h; green fluorescence (SYTO-9) represents live cells, and red fluorescence (PI) indicates dead cells. The GO group scale bar: 10 μm and the other groups scale bar: 20 μm [138]. Copyright 2022, Elsevier. (**b**) (**i**) Schematic diagram of endogenous/photodynamic synergistic antimicrobial hydrogel microneedles for diabetic wound treatment. (**ii**) From top to bottom is live/dead staining (Scale bar = 200 μm) and SEM images (Scale bar = 1 μm) of *E. coli*. (**iii**) From top to bottom is live/dead staining (Scale bar = 200 μm) and SEM images (Scale bar = 1 μm) of *S. aureus* [139]. Copyright 2023, Elsevier. (**c**) Schematic illustrations of the H2-releasing MN (AB@MSN-GO/Co^2+^-MN) for diabetic infected wound treatment [142]. Copyright 2024, Elsevier.

For antibacterial agent selection, early studies predominantly relied on antibiotic loading strategies, but the emergence of drug-resistant strains has shifted research toward non-antibiotic approaches. Metallic nanoparticles (e.g., AgNPs and ZnONPs) exert synergistic antibacterial effects through multiple mechanisms: Ag^+^ induces membrane rupture by binding to bacterial thiol proteins while inhibiting biofilm formation, whereas ZnO generates ROS to damage bacterial DNA [74]. Notably, smaller nanoparticles exhibit superior antibacterial efficiency compared to larger counterparts due to their surface-area-dependent ion release kinetics. Recently, gas therapy has emerged as a novel approach for biofilm eradication. Ma et al. [138] developed a NO-releasing microneedle using S-nitrosoglutathione (GSNO) as a prodrug, which triggers NO cascade release in the mildly acidic wound microenvironment. The released NO reacts with ROS to generate peroxynitrite (ONOO^−^), disrupting bacterial membrane phospholipid bilayers, while S-nitrosylation modifications inhibit DNA repair enzymes (e.g., RecA). Experimental results demonstrated significant bacterial load reduction in infected wounds without systemic toxicity (Figure 6a). However, long-term cytotoxicity of metallic nanoparticles (e.g., AgNP-induced mitochondrial membrane potential collapse) and dose-dependent vasodilation effects of NO require optimization through pharmacokinetic modeling.

To address the potential toxicity and inefficacy caused by uncontrolled drug release, recent studies innovatively exploit dynamic changes in the biofilm microenvironment (e.g., pH and bacterial enzymes) or modulate temperature, light, and electricity to achieve intelligent drug release for bacterial suppression. For instance, Wang et al. [139] designed a Gel/PL@F IIITA hydrogel microneedle patch that generates localized hyperthermia under 808 nm laser irradiation, disrupting Fe^3+^-TA coordination bonds to enable pulsatile NO release. Concurrently, the photothermal effect destabilizes biofilm structural integrity, achieving dual-mechanism bacterial inhibition and accelerating diabetic wound healing (Figure 6b). To tackle diabetic wound repair challenges, Li et al. [140] developed a self-powered microneedle system (TZ@mMN-TENG) that integrates tannin-modified ZnONPs with a triboelectric nanogenerator (TENG) to convert biomechanical energy into ES. In vitro studies revealed that Zn^2+^ released from ZnONPs achieved >99% bactericidal rates against *Staphylococcus aureus* and *Escherichia coli* via membrane potential disruption and ROS generation, while tannin’s phenolic hydroxyl groups conferred exceptional antioxidant capacity (>85% scavenging rate). Furthermore, ES synergized with Zn^2+^ to activate VEGF pathways, increasing the vascular density of CD31^+^ by 3.7-fold and enhancing collagen alignment by 82%. This study validates that electroresponsive MNs offer an efficient solution for diabetic wound repair through antibacterial–anti-inflammatory–proangiogenic cascades.

During antibacterial microneedle fabrication, patch size and shape can be fine-tuned to match infected wound characteristics [141], thereby enhancing clinical applicability. Post-removal, prolonged closure of microchannels increases infection risks, necessitating strategies to minimize closure time during manufacturing.

#### 3.2.3. Anti-Inflammatory and Antioxidant

The dynamic regulation of ROS balance during the inflammatory phase represents a central challenge in microneedle design. While neutrophil-derived ROS exhibit inherent antimicrobial activity, excessive ROS levels activate the NLRP3 inflammasome, leading to overproduction of IL-1β and TNF-α [143]. Unlike hydrogels and nanofibers limited to passive diffusion, MNs penetrate epidermal barriers or biofilms to directly deliver anti-inflammatory/antioxidant agents to the dermis or inflammatory foci (penetration depth: 500–1000 μm) [144]. Dissolvable and hydrogel MNs are the most frequently utilized types during this phase.

MNs enable sequential anti-inflammatory and antioxidant release through stratified drug loading. Yin et al. [145] developed a multifunctional magnesium organic framework along with a microneedle patch. The tip layer, composed of magnesium organic frameworks (Mg-MOFs) and a γ-polyglutamic acid (γ-PGA) hydrogel, achieves biphasic release: Rapid Mg^2+^ release in the initial phase (cumulative release of 81.4% in 30 min) suppresses pro-inflammatory cytokines and activates cadherin signaling pathways to enhance fibroblast migration (40% improvement) and endothelial tubulogenesis, accelerating inflammation resolution. Sustained GA release in later phases scavenges excessive ROS (>85% clearance) and inhibits NF-κB pathways to block inflammatory cascades. The backing layer, fabricated from graphene oxide–silver nanocomposites (GO-Ag), provides prolonged antibacterial efficacy (>99% inhibition against *Staphylococcus aureus* and *Escherichia coli*) to prevent secondary infections. In diabetic mouse models, the MN-MOF-GO-Ag group demonstrated significantly superior wound healing rates compared to monotherapy groups, validating the efficacy of this stratified sequential release strategy. Although localized hyperthermia provides potent bactericidal efficacy, it risks damaging neo-tissues at wound margins. Implementing gentle photothermal protocols with prolonged irradiation duration can compensate for reduced power density while minimizing adverse thermal effects.

To optimize ROS modulation, researchers have developed photothermal/gas combination therapies beyond conventional hydrogel-based materials and antioxidants. Tao et al. [142] engineered an innovative AB@MSN-GO/Co^2+^-MN composite microneedle with photothermal-responsive H_2_ release (Figure 6c). The microneedle layer contains ammonia borane (AB) encapsulated in mesoporous silica, enabling controlled H_2_ release in the moist wound microenvironment to selectively neutralize toxic ROS (·OH and ONOO^−^ scavenging >90%) and promote macrophage M2 polarization, coupled with NF-κB pathway downregulation. The GO/Co^2+^ backing layer generates localized hyperthermia under 808 nm laser irradiation, achieving rapid debridement via bacterial membrane disruption (>99% bactericidal rates against *S. aureus* and *E. coli*) and biofilm EPS degradation. Furthermore, H_2_ diffusion reduces oxygen-containing groups in GO, releasing Co^2+^ to activate HIF-1α pathways and synergizing with H_2_ to enhance endothelial tubulogenesis. This dynamic integration of gas release (microneedle layer) and photothermal/Co^2+^ release (backing layer) establishes an “antibacterial-anti-inflammatory/antioxidant-proangiogenic” cascade therapy. The effective regulation of ROS is crucial for controlling wound inflammation. However, an ideal ROS range during wound healing has not yet been determined. Thus, microneedle technology requires further investigation to improve ROS concentration control and facilitate clinical translation.

Effective ROS regulation is critical for wound inflammation control. However, current research lacks quantitative standards for ROS thresholds across healing stages, and existing microneedle systems exhibit limited ROS-responsive sensitivity (detection limit > 10 μM), hindering dynamic feedback regulation [146]. Future advancements may focus on intelligent responsive MNs [147], such as incorporating ROS-cleavable thioketal linkages into polymer backbones or integrating graphene quantum dots as in situ ROS sensors.

#### 3.2.4. Tissue Regeneration

Tissue regeneration involves the precisely coordinated processes of angiogenesis, ECM remodeling, and re-epithelialization [148]. The three-dimensional topological structure of MNs (porosity > 80%) provides a biomimetic scaffold for cell migration, while their hierarchical drug-loading capacity enables spatiotemporal release of growth factors [149]. Hydrogel MNs and dissolvable MNs are commonly employed during the tissue regeneration phase.

Beyond regenerative materials or growth factor loading, gaseous small molecules (e.g., NO and hydrogen (H_2_)) exhibit unique roles in tissue regeneration, including angiogenesis promotion, inflammation modulation, and ROS scavenging. Despite challenges posed by their high diffusivity, short half-life, and dose-dependent effects, MNs with penetration depths of 500–1000 μm enable direct delivery of gaseous precursors (e.g., GSNO) to the dermis or regenerative core, minimizing superficial diffusion losses. For instance, Liu et al. [150] designed a responsive dual-layer MN system for NO/O_2_ delivery. The outer MnO_2_ layer decomposes H_2_O_2_ in high-ROS wound environments to release O_2_, significantly reducing ROS levels, alleviating hypoxia from vascular damage and promoting endothelial cell migration and proliferation. The inner NO donor releases NO via S-N bond cleavage, which not only activates the VEGF signaling pathway to enhance endothelial migration and proliferation but also upregulates neurogenic genes (BDNF and TrkB) to improve neuronal survival and axonal extension through the PI3K-AKT-mTOR pathway. Experimental results demonstrated that the MN-O_2_/NO group achieved a remarkable wound healing rate of 95%. However, the biological safety of MnO_2_ decomposition products should be assessed to avoid Mn^2+^ accumulation and neurotoxicity after long-term use.

**Figure 7:**
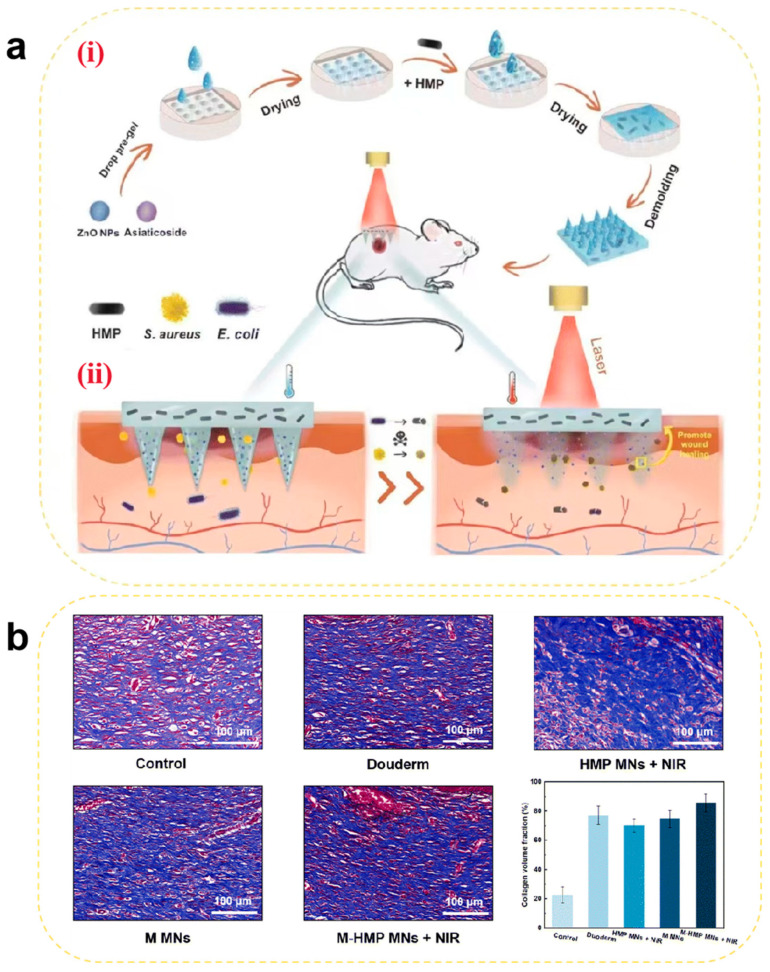
(**a**) Schematic illustration of (**i**) the preparation of M-HMP MNs and (**ii**) M-HMP MNs for wound healing. (**b**) Collagen deposition was analyzed by Masson’s trichrome staining after 9 days. Data are shown as the mean ± SD (*n* = 3) [151]. Copyright 2023, The Royal Society of Chemistry.

Traditional Chinese medicine (TCM) extracts with potent pro-proliferative effects and biosafety have also been utilized in tissue repair. Essential trace metal ions (e.g., Mg^2+^, Zn^2+^, and Cu^2+^) not only suppress bacterial infections and inflammation but also facilitate cellular proliferation and tissue regeneration. For example, Cai et al. [151] developed a sustained-release MN system co-loaded with asiaticoside (a TCM extract) and ZnO nanoparticles that were integrated with PTT. In diabetic rat models, asiaticoside-loaded MNs alleviated chronic inflammation by suppressing pro-inflammatory factors (e.g., TNF-α and IL-1β) and activated fibroblast proliferation to enhance type III collagen synthesis and accelerate re-epithelialization. Through dual mechanisms of anti-inflammation and pro-collagen synthesis, this approach disrupts the “chronic inflammation-delayed repair” vicious cycle in diabetic wounds. Meanwhile, ZnO nanoparticles release Zn^2+^ in the acidic wound environment to disrupt bacterial membranes while activating the VEGF signaling pathway to enhance endothelial cell migration. This dual functionality—antibacterial and pro-angiogenic—addresses both infection and hypoxia. Combined with photothermal-assisted targeted delivery, the system achieves synergistic healing effects, offering a novel therapeutic strategy for diabetic chronic wounds (Figure 7).

However, individual variations in skin thickness may lead to inconsistent MN penetration depths, causing heterogeneous spatial distribution of active ingredients. Furthermore, most MNs for tissue regeneration are fabricated using fixed-shape molds. While this ensures simplicity and reproducibility, it limits their adaptability to irregular wound geometries [152]. Emerging 4D printing technology may provide solutions by utilizing temperature-responsive materials (e.g., PNIPAM) to achieve autonomous shape adaptation of MN arrays. Additionally, integrating single-cell sequencing with MNs could elucidate cellular heterogeneity in tissue regeneration and advance personalized therapeutic strategies.

## 4. Electrospun Nanofibers

Electrospinning is a fiber fabrication technique driven by a high-voltage electric field that enables the solid deposition of polymer solutions or melts onto a collector, producing fibers ranging from micrometers to nanometers in diameter [12]. Nanofibers generated through electrospinning technology offer advantages, including a high specific surface area, porosity, ease of manufacturing, a low cost, excellent biocompatibility, and biodegradability, and are widely studied for use in wound dressings. Many electrospun nanofibers composed of biomaterials demonstrate good adhesion to the skin, while their porous structure enables rapid permeation of nutrients, gases, and other substances into the tissue. Additionally, they mimic the ECM to enhance cell proliferation, demonstrating excellent wound healing capabilities [12].

### 4.1. Preparation

Electrospinning can be classified based on solution characteristics and preparation methods into co-electrospinning [153], coaxial electrospinning [154], and emulsion electrospinning [155]. An electrospinning device typically comprises four components: a high-voltage power supply, a syringe pump, a spinneret, and a grounded metal collector. Under the influence of an applied electric field, the electrostatic repulsion exerted on the polymer solution or molten polymer surpasses the surface tension, forming a conical shape (Taylor cone) at the tip of the spinneret. As the voltage increases, the droplet elongates into a jet that evaporates or cools, leading to the deposition of fibers onto the collector [154].

### 4.2. Classification

Electrospun nanofibers can be categorized into natural polymer-based, synthetic polymer-based, and composite polymer-based types based on their composition.

Natural polymer-based electrospun nanofibers, composed of biopolymers such as gels, CS, and collagen, are widely employed in wound repair due to their exceptional biocompatibility and ECM-mimicking properties. For instance, during hemostasis, collagen fibers enhance wound adherence through fibrinogen adsorption to reduce blood exudation. In the antibacterial phase, the cationic nature of CS fibers directly disrupts bacterial membranes. During the anti-inflammatory and antioxidant phases, HA fibers suppress the NF-κB pathway to reduce IL-6 and TNF-α release [156]. For tissue regeneration, collagen fibers guide directional fibroblast migration and collagen deposition. Nevertheless, the limited fluid absorption capacity of natural materials challenges their use in highly exudative ulcers, often requiring multi-layer dressing designs. For example, Yang et al. [157] developed a CS–cotton fiber composite dressing, which can significantly enhance fluid absorption efficiency through a biomimetic tree-like moisture-wicking structure.

Electrospun nanofibers fabricated from synthetic polymers such as poly(vinylidene fluoride-co-hexafluoropropylene) (PVDF-HFP), polyacrylonitrile (PAN), and PVA via electrospinning exhibit phase-specific functionalities in wound healing. For hemostasis, blended PAN/PVDF-HFP fibrous networks with high porosity rapidly absorb blood to form physical barriers [158]. In antibacterial applications, PVA fibers achieve uniform antimicrobial agent dispersion through electrospinning for efficient bactericidal effects. During the anti-inflammatory, antioxidant, and tissue regeneration phases, these fibers can be functionalized with antioxidants or growth factors (e.g., VEGF and EGF) to reduce ROS levels, suppress inflammation, or induce targeted vascularization and epidermal regeneration. Despite their stable mechanical properties, prolonged degradation cycles may trigger foreign body reactions, and residual monomer cytotoxicity requires rigorous evaluation. For instance, HOF@PVDF-HFP composite fibers developed by Fudan University (China), incorporating photoactive hydrogen-bonded organic frameworks (HOFs), eliminate 99% of pathogens within 30 min under ambient light, yet prolonged use may inhibit fibroblast activity [159].

Composite polymer-based electrospun nanofibers, engineered through natural/synthetic polymer blending or functionalization, integrate bioactivity with mechanical robustness, making them a prevalent choice in wound healing. For hemostasis, quaternized N-halamine CS (CSENDMH)-PVA nanofibrous membranes synergize charge-mediated coagulation and mechanical occlusion, demonstrating superior hemostatic efficacy [160]. In the antibacterial, anti-inflammatory, and antioxidant phases, CS-PVA composite fibers co-loaded with mupirocin (MP) and cerium oxide nanoparticles (CeNPs) exhibit dual antimicrobial and ROS-scavenging capabilities [161]. For tissue regeneration, carboxymethyl guar gum (CMGG)–reduced graphene oxide (rGO)–PVA composite fibers mimic the porous architecture of the native ECM, markedly enhancing complex tissue regeneration [162]. However, their fabrication involves intricate processes and elevated production costs.

Secondary functionalization of natural, synthetic, or composite polymeric nanofibers yields biofunctionalized and biomineralized polymeric nanofibers, representing advanced wound dressing platforms. Biofunctionalized polymeric nanofibers acquire targeted bioactivities (e.g., antibacterial, anti-inflammatory, antioxidant, and pro-angiogenic) through chemical modification or biomolecule conjugation. As exemplified by Homaeigohar et al. [163], L-carnosine functionalization of PAN nanofibers significantly enhanced the viability of fibroblasts (L929) and endothelial cells (HUVECs), accelerating angiogenesis and wound re-epithelialization. Biomineralized polymeric nanofibers are engineered via deposition of inorganic minerals onto/within fiber matrices. In the work by Haider et al. [164], alkali lignin incorporation into cellulose nanofibers served dual functions: acting as reducing agents for in situ synthesis of antibacterial CuO nanoparticles while concurrently conferring potent antioxidant activity.

### 4.3. Application in Different Stages of Wound Healing

#### 4.3.1. Hemostasis

Electrospun nanofibers achieve physical hemostasis through their three-dimensional porous topological structure, which mimics the morphology of natural fibrin networks. Their high specific surface area enables efficient platelet adsorption and activation of the contact coagulation pathway [154]. Critical coagulation factors (e.g., FXII) undergo conformational changes on the fiber surface, triggering the intrinsic coagulation cascade. Studies have demonstrated that CS-based nanofibers accelerate thrombin generation rates through electrostatic interactions between their positively charged surfaces and phosphatidylserine on erythrocyte membranes [165].

**Figure 8:**
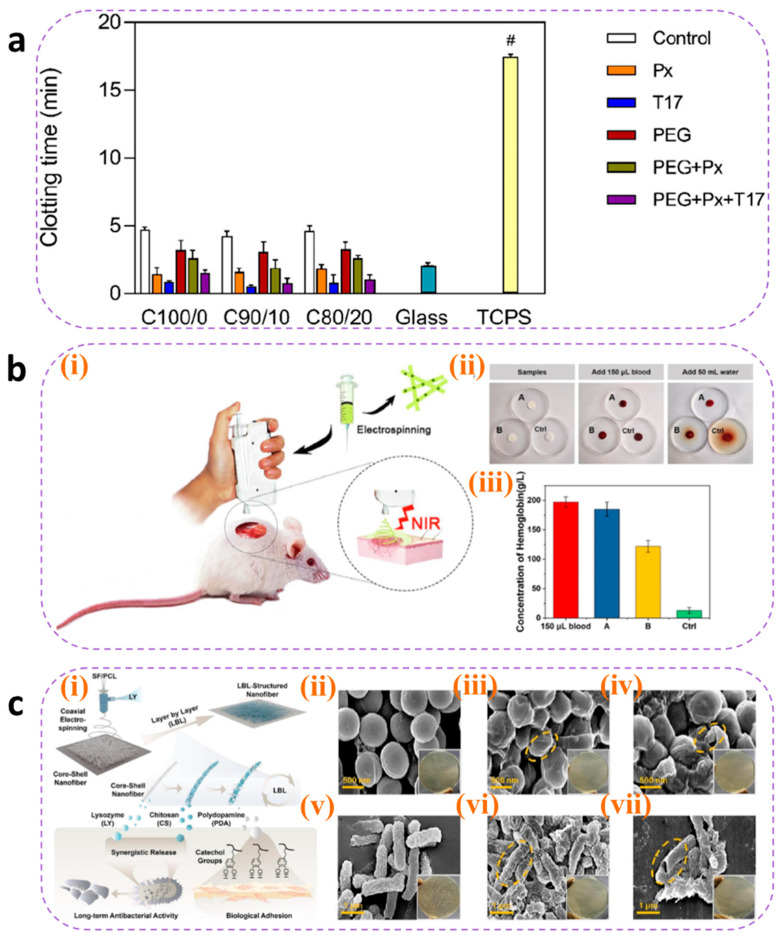
(**a**) Clotting time of recalcified plasma in the presence of unloaded (control) and loaded C100/0, C90/10, and C80/20 mats. Glass and TCPS were used as the positive and negative controls, respectively. Statistical significance was determined via the Kruskal–Wallis test and by conducting multiple comparisons between the different mat functionalizations and control groups.; it is indicated by # and means: C100/0: T17 vs. TCPS, * *p* = 0.0401; C90/10: T17 vs. TCPS, ** *p* = 0.0076; and C80/20: T17 vs. TCPS, * *p* = 0.0169 [166]. Copyright 2022, Elsevier. (**b**) (**i**) Schematic diagram of CuS composite nanofibers via in situ electrospinning with a portable device for simultaneous hemostasis and ablation of superbacteria. (**ii**) The photographs of the blood-clotting process. (**iii**) The concentration of hemoglobin in the blood clots on the materials. A group: nanofiber membranes with a 6 s electrospinning time with a thickness of approximately 400 μm; B group: nanofiber membranes with a 4 s electrospinning time and the thickness of approximately 250 μm; the control group is medical gauze [168]. Copyright 2020, Elsevier. (**c**) (**i**) Schematic route of core–shell nanofibers decorated by LBL self-assembly for the application of long-term antibacterial activity and biological adhesion. FE-SEM images of *S. aureus* treated with (**ii**) PBS (Scale bar = 500 nm), (**iii**) (CS/PDA)5 (Scale bar = 500 nm), and (**iv**) (CS/PDA)10 (Scale bar = 500 nm)and *E. coli* treated with (**v**) PBS (Scale bar = 1μm), (**vi**) (CS/PDA)5 (Scale bar = 1μm), and (**vii**) (CS/PDA)10 (Scale bar = 1μm) for 24 h, and yellow circle indicated the morphology changes in cells. The inset photos exhibited the agar plates of *S. aureus* and *E. coli* colonies for 24 h. Error bars represented the standard deviation (*n* = 3) [169]. Copyright 2023, Elsevier.

Thrombin, coagulation factors, and hemostatic peptides act as pivotal hubs in hemostatic pathways. When loaded onto nanofibers mimicking the ECM structure, their inherent compatibility synergistically enhances the hemostatic efficacy of wound dressings. Teixeira et al. [166] incorporated Pexiganan and Tiger 17 peptides (both containing lysine residues) into cellulose nanocrystal-reinforced PVA nanofiber membranes. These peptides positively influenced the intrinsic coagulation cascade, significantly reducing the clotting time compared to controls (Figure 8a).

Metal ions play critical roles in blood coagulation through synergistic mechanisms, with commonly utilized hemostatic ions including Ca^2+^, Zn^2+^, Cu^2+^, and Fe^3+^ [167]. Elevated intracellular Ca^2+^ concentrations trigger platelet deformation, α-granule release (containing ADP and factor V), and glycoprotein IIb/IIIa receptor activation to promote platelet aggregation. Coagulation factor IV and Ca^2+^ are indispensable for prothrombin (factor II) conversion to thrombin (factor IIa). Zn^2+^ enhances ADP-induced platelet aggregation by modulating membrane proteins (e.g., P2Y12 receptors) and stabilizes fibrin clots through fibrinogen binding, increasing mechanical strength by 15% (tensile strength). Cu^2+^ serves as a cofactor for factors V and VIII, facilitating prothrombinase complex assembly, while lysyl oxidase (LOX)-catalyzed fibrin α-chain cross-linking enhances clot stability. Fe^3+^, as the core component of hemoglobin, indirectly supports platelet metabolic activity by maintaining erythrocyte oxygen transport capacity. Liu et al. [168] developed a portable electrospinning device for synthesizing CuS composite nanofibers deposited in situ on wounds. Compared to conventional ex situ deposition, this approach improved nanofiber–wound adhesion. Concurrently, Cu^2+^ released from CuS nanoparticles activated coagulation factors, reducing the clotting time to less than 6 s (Figure 8b). However, the amount of metal ions added should be reasonably controlled to avoid excessive concentrations causing cytotoxicity.

However, in heavily bleeding wounds, fiber pores are prone to blood clogging, impairing deep hemostasis. Additionally, the degradation rate of nanofibers often mismatches coagulation demands—rapid degradation may lead to hemostatic failure, while slow degradation hinders subsequent tissue regeneration. To address this, researchers have designed tunable degradation composite materials using gradient cross-linking methods by adjusting the cross-linking density to synchronize degradation cycles with coagulation requirements. Alternatively, responsive nanofibers could be developed by incorporating thrombin-sensitive peptide segments to enable on-demand release of hemostatic factors.

#### 4.3.2. Antibacterial

The antibacterial properties of electrospun nanofibers originate from the synergistic effects arising from their hierarchical structural design and functional loading. The high porosity and nanoscale pore dimensions of nanofibers can physically entrap bacteria as a barrier. When employing fiber materials such as CS, the polycaprolactone–quaternary ammonium copolymer, and PEL [170], microbial adhesion can be further inhibited through surface charge interactions. Beyond conventional antibacterial agents including metal nanoparticles [171] and antibiotics [172], selected components from TCM can be incorporated as antimicrobial payloads, which generally exhibit persistent antibacterial activity with low toxicity profiles [173]. Mouro et al. [155] developed an electrospun poly(L-lactic acid) (PLLA)/PVA/CS wound dressing incorporating Hypericum perforatum (HP) extract. The sustained release of HP extract over 72 h ensured prolonged antibacterial efficacy, while cytotoxicity assays confirmed the excellent biocompatibility of the material. Its antibacterial activity was attributed to hypericin present in HP, which demonstrated a minimum inhibitory concentration (MIC) of 2.50 mg/mL against *Staphylococcus aureus*—a common pathogen in infected wounds—with the inhibition rate reaching 93.11%. These findings demonstrate the potential of nanofibrous dressings loaded with antibacterial TCM components to serve as alternatives to traditional dressings in the management of infected chronic wounds. For instance, Wu et al. [169] fabricated core–shell structured SF/polycaprolactone nanofibers using layer-by-layer (LBL) self-assembly technology. This design encapsulated lysozyme (LY) within the fiber core while alternately depositing CS and PDA on the surface. LY achieved sustained release over 14 days through fiber degradation (cumulative release > 60%), whereas surface-bound CS provided rapid release via electrostatic interactions, establishing biphasic antibacterial synergy. Experimental results demonstrated remarkable long-term antibacterial activity: over 90% bactericidal efficiency within 24 h and maintaining an inhibition rate that is >85% throughout 14 days. Field-emission scanning electron microscopy (FE-SEM) revealed severe disruption of bacterial cell membranes following CS/PDA treatment. This study validates that the spatiotemporal synergistic release strategy of LY and CS not only addresses antibiotic resistance issues associated with conventional treatments but also provides an optimal dressing design that integrates biosafety, mechanical adaptability, and prolonged antibacterial functionality for chronic infected wounds (Figure 8c).

Although the high specific surface area of nanofibers provides an ideal platform for loading antimicrobial agents, simple physical encapsulation often leads to burst release, reducing antimicrobial efficacy and causing localized cytotoxicity (e.g., high concentrations of Ag^+^ may penetrate cells and damage DNA). To address this, coaxial electrospinning for core–shell fiber fabrication or nano-encapsulation techniques have been employed to achieve zero-order release of antimicrobial agents. Additionally, molecular switches such as MMP-9-sensitive peptides can be utilized to trigger antibiotic release at infection sites through biofilm-responsive mechanisms, effectively resolving uncontrolled release kinetics.

#### 4.3.3. Anti-Inflammatory and Antioxidant

Anti-inflammatory and antioxidant activities are tightly linked through the “inflammation-oxidation axis” in wound healing: inflammation induces oxidative stress, and oxidative stress exacerbates inflammation. Pro-inflammatory cytokines activate NADPH oxidase, leading to excessive ROS production, lipid peroxidation, and DNA damage. These ROS further activate the NF-κB pathway, promoting the secretion of pro-inflammatory cytokines such as IL-6 and IL-8, thereby forming a vicious “inflammation-oxidation” cycle. Consequently, coordinated regulation of immune cell phenotypes/quantity, inflammatory cytokine expression levels, and ROS concentration is critical for effective wound healing.

**Figure 9:**
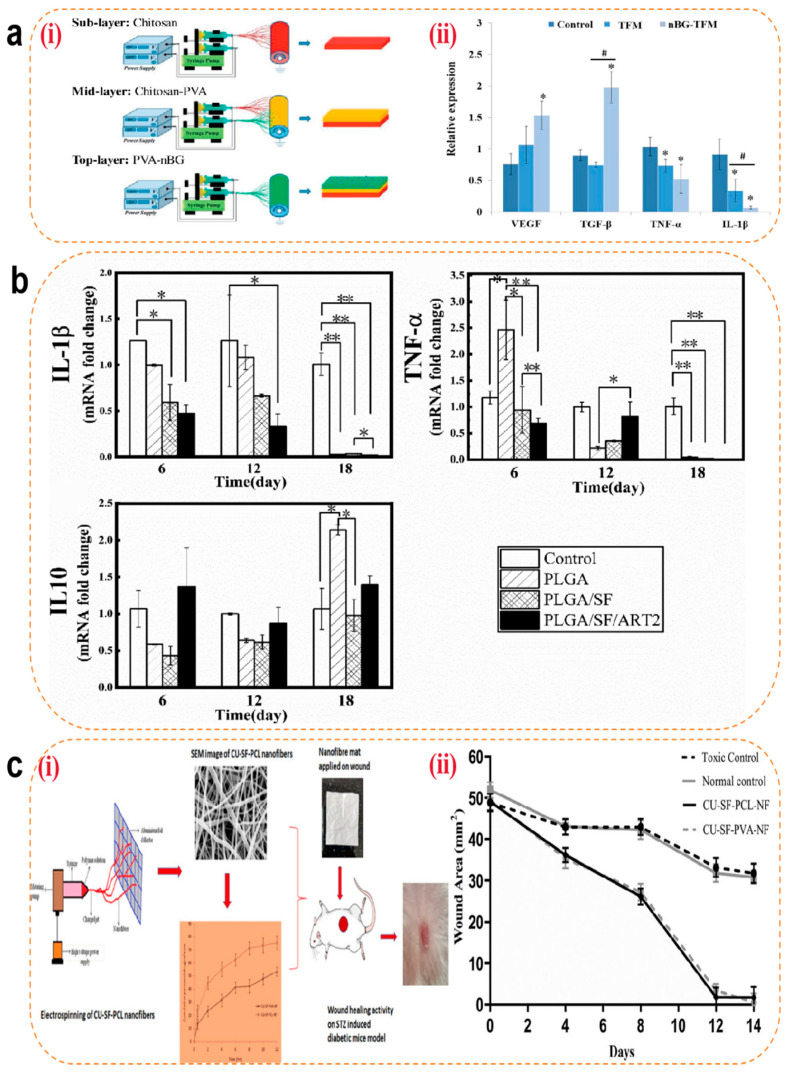
(**a**) (**i**) Schematic of nBG-TFM fabricated by sequential electrospinning. (**ii**) Related gene expression of growth factors (VEGF and TGF-β) and inflammatory cytokines (TNF-α and IL-1β) in diabetic wounds with different treatments analyzed by RT-PCR. * *p* < 0.05 (vs. control) and # *p* < 0.05 indicate significance [174]. Copyright 2019, Elsevier. (**b**) Level of inflammation-related gene expression in wound regeneration with different treatments: * *p* < 0.05 and ** *p* < 0.01; data represent means ± SD (*n* = 3) [179]. Copyright 2021, Elsevier. (**c**) (**i**) Schematic diagram of electrospun nanofibers loaded with curcumin for diabetic wound treatment. (**ii**) Wound healing rate for all the groups (normal control/toxic control group vs. formulation groups (placebo, CU-SF-PCL-NF, and CU-SF-PVA-NF) *p* < 0.001) [182]. Copyright 2021, Elsevier.

Electrospun nanofibers can spatially regulate inflammatory responses, with their unique functional layering strategy demonstrating synergistic advantages in the anti-inflammatory and antioxidant phases to optimize wound healing. For example, Chen et al. [174] designed a trilayer nanofibrous composite membrane (nBG-TFM) via sequential electrospinning. The spatially structured membrane consists of a CS bottom layer, a CS/PVA middle layer, and a PVA/nanobioglass (PVA/nBG) top layer. The CS layer rapidly activates platelets and erythrocytes via charge interactions to reduce bleeding and bacterial infection, mitigating inflammation at its source. The PVA middle layer maintains a moist environment to minimize oxidative stress. The nBG top layer continuously releases Ca^2+^ and Si^4+^, activating fibroblasts to secrete VEGF and TGF-β, thereby promoting angiogenesis, collagen synthesis, and inflammation resolution. In a diabetic chronic wound model, nBG-TFM significantly downregulated the pro-inflammatory factors TNF-α and IL-1β while enhancing antioxidant-related gene activity (e.g., SOD1), alleviating oxidative microenvironments and achieving dynamic anti-inflammatory–antioxidant balance through spatial functional layering (Figure 9a). However, it is important to ensure that the multi-layer structure is stable and does not separate in a moist wound environment, which could affect functional stability.

Certain traditional Chinese medicinal components—such as aloe vera [175], honey [176], astragaloside IV [177], and artemisinin—exhibit immunomodulatory and anti-inflammatory properties [178] and are commonly used as anti-inflammatory agents to mediate wound healing. Peng et al. [179] embedded artemisinin (ART) into poly(lactic-co-glycolic acid)/SF (PLGA/SF) fibers, resulting in a membrane with stable and sustained release. Cumulative ART release reached 69% after three weeks. The released ART inhibited inducible nitric oxide synthase (iNOS) by blocking macrophage transcriptional signaling pathways, thereby reducing the release of inflammatory cytokines (TNF-α and IL-6) and the mediator nitric oxide (NO). Experimental results demonstrated that ART-loaded fibrous membranes promoted macrophage polarization from the M1 phenotype to the M2 phenotype, significantly decreasing IL-1β and TNF-α levels while increasing IL-10 expression (Figure 9b).

Antioxidants scavenge free radicals at wound sites, mitigating tissue damage [180] with high efficiency in ROS clearance. Natural polyphenolic compounds—such as tea polyphenols, resveratrol, curcumin, ferulic acid, anthocyanins, and flavonoids [181]—are widely used in wound dressings. Electrospun nanofibrous dressings loaded with antioxidants prevent burst release through optimized material and structural design, ensuring effective antioxidant performance during the inflammatory phase. For instance, Agarwal et al. [182] fabricated curcumin-loaded PCL/PVA-SF composite nanofibrous membranes (CU-SF-PCL-NF and CU-SF-PVA-NF) via electrospinning. These membranes accelerated diabetic wound healing through dual anti-inflammatory and antioxidant mechanisms: SF provided a biomimetic ECM structure, while PVA/PCL balanced hydrophilicity/hydrophobicity for sustained curcumin release. In vitro studies showed that curcumin significantly suppressed the pro-inflammatory cytokines TNF-α and IL-1β (40–50% reduction) while upregulating SOD activity (1.5-fold increase) to reduce ROS accumulation. Curcumin further enhanced antioxidant defenses via Nrf2/ARE pathway activation and inhibited NF-κB signaling to attenuate inflammation, achieving synergistic anti-inflammatory–antioxidant effects. In diabetic mice, the CU-SF-PCL-NF group exhibited a 3-fold increase in healing rate, reducing the wound area to 1.74 mm^2^ (vs. 31.79 mm^2^ in controls) by day 14 (Figure 9c). However, polyphenols are prone to oxidative deactivation, significantly reducing their antioxidant activity. Current research focuses on formulating β-cyclodextrin inclusion complexes to enhance their stability and storage resistance.

#### 4.3.4. Tissue Regeneration

The majority of fibrous materials, including HA, SF, collagen [183], and PLLA, exhibit bioactivity. These materials can simulate the nanoscale architecture of the ECM through electrospinning, synergistically constructing a three-dimensional regenerative microenvironment [184]. Aligned fibers regulate cytoskeletal remodeling via mechanotransduction, guiding the directed migration of fibroblasts and promoting the arrangement of collagen fibers secreted by fibroblasts to resemble native skin tissue.

**Figure 10:**
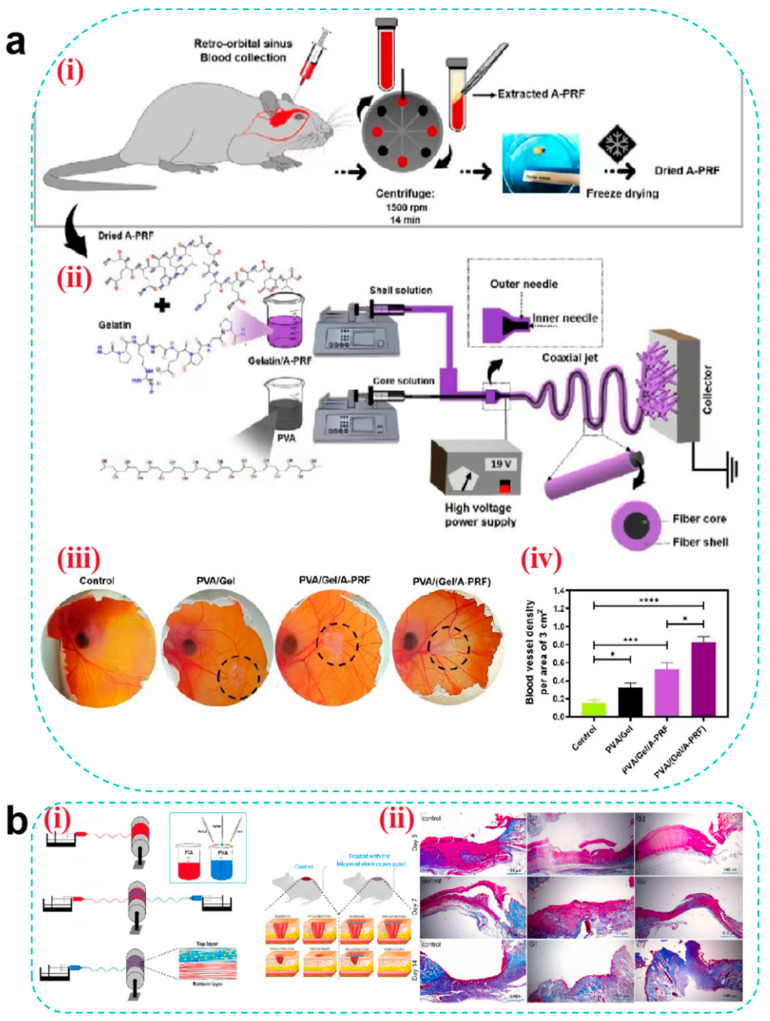
(**a**) (**i**) Extraction and preparation of dried A-PRF. (**ii**) Preparation of core–shell nanofibers by the coaxial electrospinning method. (**iii**) Angiogenic potential of nanofiber wound dressings investigated by the CAM assay. (**iv**) Blood vessel density [185]. Copyright 2022, Elsevier. (**b**) (**i**) Schematic diagram of electrospun nanofibers loaded with vitamins and silver nanoparticles for wound treatment. (**ii**) Results from Masson’s trichrome staining after 3, 7, and 14 days. Scale bar = 100 μm [187]. Copyright 2024, Elsevier.

Electrospun nanofibers have emerged as advanced bioactive delivery systems during the regenerative phase of wound healing. Curcumin-loaded nanofibers scavenge free radicals by activating the Nrf2 pathway while enhancing fibroblast proliferation. Fibrinogen-incorporated fibers accelerate hemostasis by releasing fibrinopeptide B to promote platelet aggregation [185]. Metal ion-doped fibers regulate HIF-1α and mTOR signaling pathways to stimulate neovascularization and cellular proliferation [186]. Furthermore, these nanofibers enable precise delivery of growth factors (e.g., VEGF, FGF, and TGF-β) through optimized structural designs for controlled release, achieving synergistic enhancement of angiogenesis, collagen deposition, and re-epithelialization. Tavakoli et al. [185] developed a core–shell structured nanofibrous wound dressing (PVA/(Gel/A-PRF)) using coaxial electrospinning. The PVA core provides mechanical support (tensile stress: 7.43 ± 0.38 MPa), while the gel shell incorporates advanced platelet-rich fibrin (A-PRF) to synergistically release growth factors for accelerated full-thickness wound healing. This core–shell design combines the high modulus of PVA (50–200 MPa, matching skin biomechanical requirements) with the bioactivity of Gel/A-PRF. The scaffold demonstrated controlled degradation (47.41 ± 1.97% within 7 days), synchronized with tissue regeneration kinetics to prevent premature structural collapse. Sustained release of VEGF and PDGF-AB from A-PRF over 7 days was achieved through the core–shell architecture, where VEGF activated endothelial cell migration and PDGF enhanced collagen organization. In vitro chorioallantoic membrane (CAM) assays revealed significantly higher vascular density in the PVA/(Gel/A-PRF) group compared to the controls (Figure 10a). However, there are still differences between the in vitro CAM model and the complex microenvironment in vivo, and the actual clinical significance of increased vascular density needs to be further verified.

Beyond their roles in metabolic regulation, vitamins can also promote tissue regeneration. If combined with the nanofiber delivery system, the stability and efficacy of vitamins can be significantly enhanced by structural design (e.g., core–shell protection and layered drug delivery), which can play a key role in the regeneration phase of wound healing through multi-dimensional regulation. Iranpour Mobarakeh et al. [187] developed a bilayer PCL/PVA dressing (G2) that synergistically combines vitamin B12/C with silver nanoparticles (AgNPs) for dual antimicrobial and collagen synthesis enhancement. Vitamin C directly reinforces dermal reconstruction by stimulating fibroblast proliferation and collagen production, while vitamin B12 modulates inflammatory responses and epithelial regeneration to shorten the inflammatory phase and accelerate epidermal coverage. The synergistic effect of AgNPs not only reduces the risk of infection through antimicrobial activity but also reduces the damage of ROS to regenerated tissues through an antioxidant mechanism, thus creating an ideal microenvironment for tissue regeneration. In vivo studies demonstrated that the G2 group achieved an 8 mm reduction in full-thickness wound diameter within 14 days, with histological analyses revealing superior collagen deposition density and neovascularization compared to the controls. Masson’s trichrome staining further indicated more organized collagen fiber alignment in the G2-treated wounds, suggesting that vitamin C optimizes ECM remodeling by activating collagen cross-linking enzyme systems. These findings highlight the dual advantages of vitamins during the proliferative and remodeling phases of wound healing: Vitamin C-driven collagen metabolism synergizes with vitamin B12-mediated inflammation–regeneration balance to promote efficient and orderly tissue repair, providing a theoretical foundation for developing multifunctional wound dressings (Figure 10b). However, during the preparation process, if the material layer is hydrophobic, the water-soluble vitamins should be uniformly distributed in the hydrophobic material layer through strategies such as nanoencapsulation, structure optimization, and component separation to avoid affecting the long-term activity and safety of the vitamins.

## 5. Stimuli-Responsive Wound Dressings

Although hydrogels, MNs, and electrospun nanofibers demonstrate significant advantages as advanced wound dressings in specific healing phases, wound repair remains a dynamic and complex pathological process requiring stage-specific adaptation to microenvironmental variations (e.g., pH, ROS levels, and enzymatic activity) and functional demands (e.g., hemostasis, anti-inflammation, and tissue regeneration). Current applications of these dressings are constrained by static passive release mechanisms, which fail to achieve temporal regulation, potentially leading to premature drug depletion, localized concentration imbalance, or mismatched therapeutic actions relative to healing progression. Consequently, when constructing new wound dressings such as hydrogels, MNs, and electrospun nanofibers, there is an urgent need to equip them with the property of releasing drugs intelligently according to changes in the microenvironment of the wound so as to break through the limitations of “passive intervention” and realize “on-demand treatment”. Stimuli-responsive wound dressings have emerged as a transformative solution. By incorporating functional structures that sense endogenous wound microenvironmental cues (e.g., pH, temperature, enzyme activity, redox status, or specific biomarkers) or respond to exogenous triggers (e.g., near-infrared light, electricity, magnetic fields, or ultrasound), these systems dynamically modulate drug release, mechanical properties, or bioactivity to precisely align with the heterogeneous demands of each healing phase. This biomimetic strategy not only enhances spatiotemporal therapeutic specificity but also synergistically disrupts the vicious cycle of “inflammation-oxidation-repair imbalance” through multi-mechanistic coordination, offering revolutionary solutions for managing complex wounds.

### 5.1. pH-Responsive Wound Dressings

**Figure 11:**
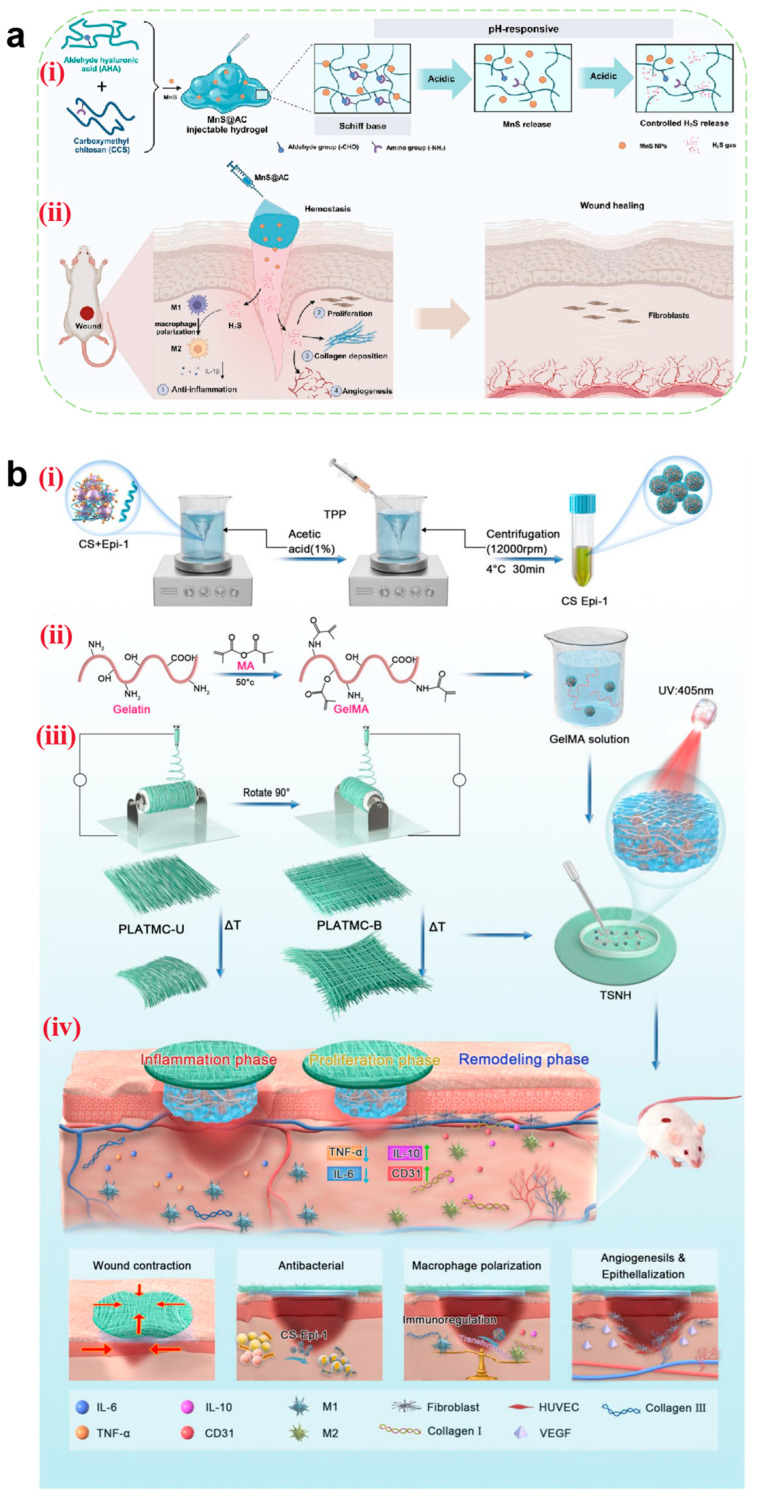
(**a**) An illustration of a pH-responsive injectable hydrogel for wound treatment. (**i**) The schematic diagram of the preparation of a pH-responsive injectable hydrogel (MnS@AC) constructed via a dynamic Schiff base reaction between aldehyde hyaluronic acid (AHA) and carboxymethyl chitosan (CCS). (**ii**) Multifunction of MnS@AC for wound healing, including anti-inflammation, proliferation, collagen deposition, and angiogenesis [193]. Copyright 2025, Elsevier. (**b**) A diagrammatic illustration of the temperature-responsive self-contraction nanofiber/hydrogel composite dressing facilitating the healing of diabetic-infected wounds. (**i**) The preparation process of CS-Epi-1 nanoparticles. (**ii**) The GelMa synthesis route and the hydrogel preparation process of the functional layer. (**iii**) The preparation process of PLAMC-R, PLAMC-U, and PLAMC-B nanofibers, as well as the preparation process of TSNH composite dressings. (**iv**) The TSNH composite dressing has the functions of mechanical contraction, antibacterial, anti-inflammatory, and promoting angiogenesis [195]. Copyright 2024, Elsevier.

The pH of the wound microenvironment typically varies across healing stages. In acute wounds, transient physiological acidosis occurs during the hemostatic and inflammatory phases due to lactate release from platelets, increased oxygen demand, and elevated pCO2 caused by impaired tissue perfusion, resulting in an acidic wound pH. Subsequently, the pH gradually rises above 7.0, and the onset of re-epithelialization during the proliferative phase may induce a downward shift, eventually stabilizing around 5.0 [188]. In contrast, the pH of chronic wounds increases to alkaline over time. For instance, in diabetic chronic wounds, their pH continues to rise after the proliferative period, eventually reaching over 8.0 and in severe cases even exceeding 9.0, due to chronic inflammation and interstitial fluids from diabetic patients flowing into the unhealed wounds as well as bacterial enzymes (e.g., urease) secreted during infection [189]. This offers the possibility of realizing on-demand release of active substances based on pH responsiveness. Two primary strategies are employed to fabricate pH-responsive dressings: (1) utilizing copolymers with ionizable or protonatable groups (e.g., alginate, PAA, and CMC), where carboxyl groups exhibit enhanced ionization under alkaline conditions, thereby increasing electrostatic repulsion and swelling rates to accelerate drug release [190], and (2) incorporating acid/base-cleavable chemical bonds (e.g., Schiff base bonds, boronate esters [191], and hydrazone linkages [192]) as cross-linking units. Sun et al. [193] developed an injectable pH-responsive polysaccharide hydrogel (MnS@AC) by dynamically cross-linking aldehyde-modified hyaluronic acid (AHA) with carboxymethyl chitosan (CCS) via Schiff base bonds and encapsulating α-phase manganese sulfide nanoparticles (MnS NPs). This system achieved on-demand hydrogen sulfide (H2S) release in acidic wound microenvironments. During the early inflammatory phase (pH 6.5), acidic conditions triggered Schiff base hydrolysis, accelerating MnS NP release. The released H2S suppressed ERK/STAT3 phosphorylation and NLRP3 inflammasome activation, promoting M2 macrophage polarization (a 40–50% reduction in IL-6 and iNOS mRNA expression) to alleviate inflammation. As healing progressed to the proliferative phase (pH 7.4), H2S release diminished, preventing high-concentration toxicity. Concurrently, Mn^2+^ released under acidic conditions remained localized in wound exudate without activating the cGAS-STING pathway, avoiding secondary inflammation from metal ions. The hydrogel’s polysaccharide components maintained a moist environment, supporting collagen deposition and angiogenesis. This multifunctional system synergized anti-inflammatory, pro-proliferative, and tissue-repair effects, providing a low-toxicity strategy for complex wound treatment (Figure 11a).

### 5.2. Temperature-Responsive Wound Dressings

Temperature, as a mild stimulus, is widely utilized in designing stimuli-responsive wound dressings. Wound temperature gradually increases during the hemostatic and inflammatory phases due to thrombin-induced thermogenesis and heightened inflammatory cell activity. However, it normalizes during subsequent phases after resolution of inflammation. Temperature-sensitive polymers, such as methylcellulose derivatives, poly(N-isopropylacrylamide) (PNIPAM), and poly(2-oxazoline) (PAOx), enable rapid phase or volume transitions at their upper critical solution temperature (UCST) or lower critical solution temperature (LCST). These materials exhibit hydrophobic contraction for hemostasis at elevated temperatures and hydrophilic swelling to enhance cell infiltration at lower temperatures [194]. Huang et al. [195] developed a bilayer temperature-responsive nanofiber/hydrogel composite dressing (TSNH) by combining a biaxially oriented poly(lactide-co-trimethylene carbonate) (PLATMC) nanofiber base layer with a gelatin methacrylate (GelMA) hydrogel functional layer loaded with epiresistin-1@CS (Epi-1@CS) nanoparticles. During the inflammatory phase of diabetic wounds (local temperature: 37.8 °C), PLATMC nanofibers contracted, with the biaxially oriented structure (PLATMC-B) generating centripetal forces (90.79% contraction rate) to mechanically approximate wound edges. In vivo studies demonstrated that the TSNH group achieved significantly higher wound closure (85.66% on day 7 vs. 10.16% in controls), reaching near-complete healing by day 14. Simultaneously, the hydrogel functional layer sustained the release of the antimicrobial peptide Epi-1 from CS nanoparticles, showing >90% inhibition against drug-resistant bacteria (e.g., MRSA). It also promoted macrophage polarization toward the M2 phenotype, effectively suppressing inflammation. This temperature-responsive system dynamically adapts to wound microenvironments through mechano-biochemical synergy, offering a multifunctional therapeutic strategy for infected diabetic wounds (Figure 11b).

### 5.3. Enzyme-Responsive Wound Dressings

**Figure 12:**
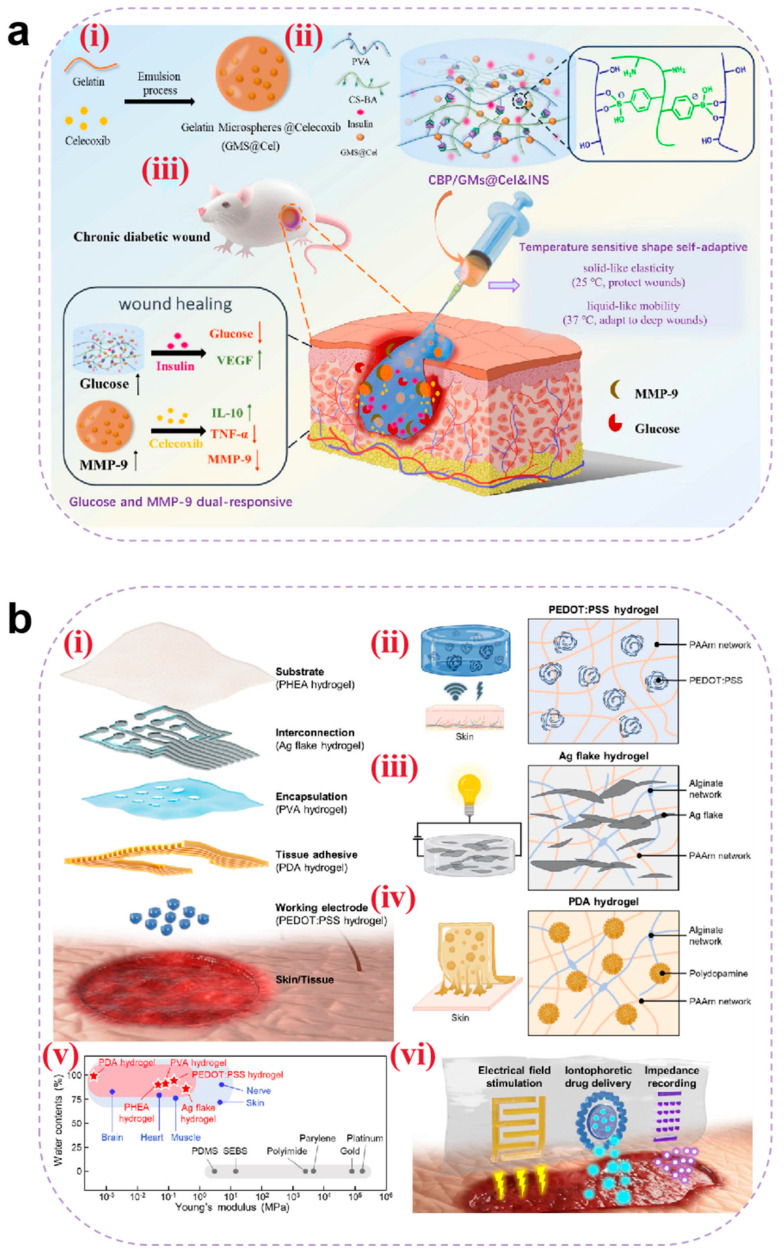
(**a**) The design strategy of glucose and MMP-9 dual-responsive shape self-adaptive hydrogels for treating chronic diabetic wound. (**i**) Preparation of gelatin microspheres containing celecoxib (GMs@Cel). (**ii**) Preparation of the CBP/GMs@Cel&INS hydrogel and the characteristics of temperature-sensitive shape self-adaptive hydrogels. (**iii**) The process of treating chronic diabetic wounds with the CBP/GMs@Cel&INS hydrogel through the glucose and MMP-9 dual-response system [199]. Copyright 2022, Elsevier. (**b**) An all-hydrogel-based electronic-skin (e-skin) patch composed of functional hydrogels. (**i**) Exploded view of a functional-hydrogel-based e-skin patch. (**ii**–**iv**) Schematic illustration of each functional hydrogel displaying its characteristics. PEDOT:PSS hydrogel with low impedance (**ii**), Ag flake hydrogel with high conductivity (**iii**), and PDA hydrogel with tissue adhesiveness (**iv**). (**v**) Water content and Young’s modulus of the functional hydrogels, biological tissues, and conventional materials used in bioelectronics. (**vi**) Representative hydrogel patterns of e-skin patch showing each application of electrical field stimulation, iontophoretic drug delivery, and impedance recording [207]. Copyright 2025, Elsevier.

Enzyme-responsive systems exhibit superior efficiency and specificity compared to other stimuli-responsive platforms. Taking MMP-8 and MMP-9 as examples, during the inflammatory phase of acute wounds, MMP-8 secreted by neutrophils transiently is upregulated to degrade type I collagen in necrotic tissues, while ROS-activated NF-κB pathway upregulates MMP-9 to remove the damaged matrix and initiate ECM remodeling [196]. Concurrently, TIMP-1 levels rise to suppress excessive MMP expression, preventing ECM over-degradation. In contrast, diabetic chronic wounds exhibit sustained MMP-8 and MMP-9 overexpression during inflammation due to hyperglycemia, advanced glycation end-product (AGE) accumulation, and dysregulated inflammation. This persistent activity disrupts the native ECM architecture, exacerbates tissue lysis, and excessively degrades basement membrane collagens (types IV, V, VII, and X), thereby impeding angiogenesis and epithelialization [197]—effects that overwhelm TIMP-1’s inhibitory capacity. To address this, researchers have engineered MMP-9-sensitive peptide sequences into gels, fibrinogen/LY complexes, and other matrices. These systems enable targeted drug release through enzymatic cleavage of peptide bonds by overexpressed wound enzymes (e.g., MMP-9 and collagenase) [198]. Zhou et al. [199] developed a hydrogel dressing via phenylboronate ester cross-linking between PVA- and PBA-modified CS, encapsulating insulin- and celecoxib-loaded gel microspheres for MMP-9 responsiveness. Elevated MMP-9 levels in diabetic wounds specifically degraded gel microspheres, triggering on-demand celecoxib release. In vitro studies demonstrated that the cumulative amount of celecoxib release reached 40% within 72 h, effectively suppressing pro-inflammatory TNF-α while upregulating anti-inflammatory IL-10, thereby shortening the inflammatory phase. MMP-9-triggered celecoxib release synergized with glucose-responsive insulin release to accelerate angiogenesis and epithelialization. In a diabetic rat full-thickness skin defect model, hydrogel-treated wounds achieved 96.68% closure by day 14, significantly outperforming commercial dressings (82.87%) and confirming its therapeutic efficacy for chronic diabetic wounds (Figure 12a).

### 5.4. ROS-Responsive Wound Dressings

ROS, highly reactive ions generated in vivo, play critical roles in regulating cellular signaling, inflammation, and proliferation. In acute wounds, ROS levels gradually increase during early healing phases due to platelet and neutrophil release, peaking transiently in the inflammatory phase via neutrophil respiratory burst, followed by a decline to physiological baseline. Conversely, in diabetic wounds, AGE accumulation enhances NADPH oxidase activity, driving persistent ROS overproduction across all healing phases [200], which markedly impedes wound repair. ROS-responsive dressings employ dynamic covalent networks (e.g., disulfide bonds, Schiff base bonds, and boronate esters) that undergo ROS-triggered bond cleavage to accelerate drug release. Wang et al. [201] designed a ROS-responsive hydrogel (BI-AuLA) based on lipoic acid (LA) supramolecular polymers, incorporating dynamic disulfide bonds, gold nanostars (AuNSs), and bovine insulin (BI) for multimodal therapy. The hydrogel was dynamically degraded via disulfide bond cleavage under elevated ROS levels, simultaneously scavenging ROS to restore redox homeostasis and releasing bactericidal AuNSs alongside hypoglycemic BI. This dual-action mechanism enhanced vascularization and collagen deposition, demonstrating therapeutic potential for refractory diabetic wounds.

### 5.5. Glucose-Responsive Wound Dressings

Driven by insulin resistance, AGE accumulation, and microangiopathy, diabetic wounds exhibit sustained local glucose elevation, establishing a “hyperglycemic vicious cycle” [202]. This pathological context underscores the significant therapeutic potential of glucose-responsive wound dressings. Current systems include glucose oxidase (GOx) [203], concanavalin A (Con A) [204], and PBA-based platforms. While GOx and Con A suffer from environmental sensitivity, a short shelf life, and immunogenicity, PBA-based dressings offer low toxicity, stability, and biocompatibility. PBA reversibly binds to 1,2-diol-containing compounds (e.g., glucose) via phenylboronate ester bonds. In hyperglycemic diabetic wounds, competitive glucose binding disrupts these bonds, enabling controlled drug release. Liu et al. [205] engineered a glucose-responsive self-healing bilayer microneedle patch (SDDMN-POGa) using dynamic phenylboronate bonds. Under hyperglycemia conditions, PBA–glucose binding triggered rapid insulin and biomimetic antibacterial agent (POGa) release. POGa, mimicking hemoglobin, employed a “Trojan horse” strategy to disrupt bacterial iron metabolism, suppressing multidrug-resistant pathogens. In vivo, SDDMN-POGa upregulated CD31 expression (angiogenesis) and reduced TNF-α (anti-inflammation) levels, achieving 99.6% wound closure by day 20 versus 87.5% in the controls. Reversible phenylboronate bonds enabled cyclic drug release under alternating glucose levels, mitigating overdose risks and offering a precise, antibiotic-free strategy for diabetic wound care.

### 5.6. Electrostimuli-Responsive Wound Dressings

Electroconductivity, an inherent skin property, enables conductive materials (e.g., PPy, PANI, and graphene/SF films) to induce cell membrane depolarization under external electric fields, activating voltage-gated Ca^2+^ channels to enhance cell migration and collagen synthesis [206]. Shin et al. [207] developed an electroresponsive hydrogel-based electronic skin patch (e-skin patch) by combining ES with iontophoresis for accelerated healing. The patch comprises low-impedance poly(3,4-ethylenedioxythiophene)/polystyrene sulfonate (PEDOT:PSS) hydrogel electrodes, highly conductive silver interconnects, and PDA tissue adhesives. ES significantly enhanced human dermal fibroblast migration and upregulated VEGF/TGF-β expression (angiogenesis), achieving 89% wound closure by day 12 versus 74% in the controls. Concurrently, iontophoresis delivered EGF to the dermis within 30 min at 0.3 mA/cm^2^, outperforming passive diffusion. Integrated impedance mapping enabled real-time healing monitoring, establishing a closed-loop theranostic platform for intelligent wound management (Figure 12b).

### 5.7. Multistimuli-Responsive Wound Dressings

Single-stimulus-responsive dressings often fail to achieve precise spatiotemporal drug delivery for optimal therapeutic outcomes. Dual/multi-stimuli-responsive systems enable synchronized control over active substance release, addressing dynamic demands across healing stages.

Heavily infected diabetic chronic wounds have high blood glucose concentrations, and a high glucose environment exacerbates bacterial infections, which are metabolized by bacteria to produce lactic acid and acetic acid, leading to the formation of an acidic microenvironment. Based on this, Zhang et al. [208] engineered a pH/glucose dual-responsive hydrogel (OPCP) via Schiff base reactions and PBA groups for smart proanthocyanidin (PA) delivery. OPCP demonstrated pH-dependent PA release (85.15% at a pH of 6.5 vs. 71.86% at a neutral pH) and glucose-responsive release (87.34% under high-glucose conditions vs. 61.87% without glucose), confirming synergistic dual-response behavior. In diabetic rat models, OPCP accelerated wound closure (a 40% improvement by day 14) via ROS scavenging, TNF-α suppression, and angiogenesis promotion, offering a localized dual-responsive strategy for type 2 diabetic foot ulcers.

## 6. Future Challenges

In recent years, wound dressings have evolved from passive traditional bandages to advanced functional systems. Hydrogels, MNs, and electrospun nanofibers—as extensively studied novel dressings—have demonstrated proven capabilities in drug delivery, hemostasis, antibacterial activity, anti-inflammation, antioxidant effects, and tissue regeneration. The crosstalk between tumor recurrence and wound healing processes—particularly in inflammation, immunosuppression, and angiogenesis—has motivated the application of wound dressings for postoperative tumor suppression. Li et al. [209] developed an Apt-GelMA hydrogel functionalized with DNA aptamers that selectively target tumor cells. The modular aptamer design allows on-demand substitution for multi-tumor applicability, while the gelatin methacrylate (GelMA) matrix provides a pro-regenerative microenvironment for healthy tissue growth. This system achieves sustained tumor recurrence inhibition and accelerated wound closure. Homaeigohar et al. [210] engineered ZnO/carnosine-functionalized polyacrylonitrile nanofibers (ZCPANs). The incorporated ZnO nanoparticles induce cancer cell apoptosis through ROS generation or Zn^2+^ ion release, effectively suppressing melanoma recurrence. This platform offers a promising strategy for post-resection therapy in skin cancer management. However, each wound dressings exhibits distinct advantages and limitations (Table 3), and several challenges persist for future development. First, from the perspective of safety, diverse materials used in novel dressings require thorough investigation of biocompatibility. Preclinical assessments must evaluate material stability and potential toxicity in complex biological environments to prevent adverse effects in vivo. Secondly, the transformation of new wound dressings from laboratory-scale synthesis to industrial production faces significant barriers. High-purity raw materials, complex cross-linking reactions, and stringent process controls escalate costs. The absence of standardized mass-production protocols results in inconsistent product quality. Solutions include selecting cost-effective materials, optimizing production workflows, establishing standardized manufacturing protocols, and enhancing supply chain integration to enable scalable production. Furthermore, how to achieve clinical transformation is also a huge challenge faced by novel wound dressings. Most novel dressings remain in early-stage clinical trials, lacking large-scale, long-term efficacy data. Combination therapies (e.g., with phototherapy or pharmaceuticals) are underdeveloped. Longitudinal follow-up studies are needed to validate long-term safety/efficacy and to establish synergistic treatment modalities. Clinical trials must incorporate wound complexity and patient heterogeneity to ensure result generalizability.

**Table 3:** Characteristics of three new wound dressings.

New Wound Dressings	Stages of Wound Healing	Similarities	Advantages	Disadvantages	Refs.
Hydrogels	Hemostasis	Adhesive and absorbs blood	Provides cooling effects to reduce perceived pain	Excessive water absorption causes volumetric expansion, potentially compressing surrounding tissues	[102,211,212]
Microneedles			Needle-shaped structures physically approximate wound edges for hemostasis	Needle fracture or improper deployment risks vascular damage	
Electrospun nanofibers			ECM-mimicking architecture enhances tissue adhesion/high surface-area-to-volume ratio accelerates hemostasis	Non-uniform fiber diameter distribution may compromise hemostatic consistency	
Hydrogels	Antibacterial	Substrate materials and drugs used are similar	Regulates wound moisture to inhibit bacterial colonization and infection	Elevated temperatures enlarge pore size, increasing bacterial translocation risks	[213]
Microneedles			Facilitates drug penetration through bacterial biofilms	Microchannel closure may trap pathogens, elevating infection susceptibility	
Electrospun nanofibers			Porous structure improves breathability and reduces bacterial proliferation	Limited mechanical strength restricts load-bearing applications	
Hydrogels	Anti-inflammatory and antioxidant	Tunable drug release properties	Macroporous design promotes gas exchange and metabolite permeation	Non-transparent nature hinders visual monitoring of wound progression	[212]
Microneedles			Disrupts hypoxia-inducing bacterial biofilms linked to chronic inflammation	Challenges in conforming to irregular wound geometries	
Electrospun nanofibers			Micropores prevent airborne particle infiltration, minimizing inflammatory risks	Scalability issues in industrial manufacturing	
Hydrogels	Tissue regeneration	Structural design can be similar to human tissue	ECM-like structure ensures high biocompatibility	Certain chemical cross-linkers exhibit cytotoxicity	[152,214]
Microneedles			Biomimetic structural design supports functional integration	Pain during dressing removal due to strong tissue adhesion	
Electrospun nanofibers			ECM-mimicking microenvironment promotes cellular proliferation and migration	Overly dense fiber networks impede cellular infiltration, delaying tissue regeneration	

## 7. Conclusions

In recent years, the use of novel wound dressings in wound treatment has been on the rise. This paper focuses on the various stages of wound healing and discusses the application of hydrogels, MNs, electrospun nanofibers, and stimulus-responsive wound dressings in wound repair, clarifying their healing mechanisms and characteristics at different stages of wound repair. However, due to the complex microenvironment of cells, their long-term efficacy in vivo remains unknown. Therefore, when designing novel wound dressings, in addition to considering their performance in terms of efficacy, biocompatibility, and mechanical strength, it is also necessary to comprehensively understand their safety when deposited in the skin and their impact on the interactions between various cellular components, thereby facilitating their rapid clinical application. The emergence of stimulus-responsive wound dressings has gradually enabled the controlled release of drugs and the monitoring and detection of wound conditions. However, there is still room for further development, as the drugs or cytokines loaded into wound dressings typically only exert their effects during specific stages of wound healing, necessitating precise temporal control to avoid adverse effects on wound healing. Additionally, developing organ-on-a-chip models and phase-sensitive materials, establishing dynamic mechanical testing standards, achieving multi-stage time-controlled drug release, and standardizing clinical translation are also worthy research topics.

## Data Availability

Not applicable.
